# Demographics of patients receiving Intravitreal anti-VEGF treatment in real-world practice: healthcare research data versus randomized controlled trials

**DOI:** 10.1186/s12886-017-0401-y

**Published:** 2017-01-19

**Authors:** F. Ziemssen, N. Feltgen, FG. Holz, R. Guthoff, A. Ringwald, T. Bertelmann, A. Wiedon, C. Korb, for the OCEAN study group

**Affiliations:** 1 Centre for Ophthalmology, Eberhard-Karls-University Tuebingen, Schleichstr. 12, Tuebingen, 72076 Germany; 2 University Eye Hospital Goettingen, Goettingen, Germany; 3 Department of Ophthalmology, University of Bonn, Bonn, Germany; 4 University Eye Hospital, Heinrich-Heine-University Duesseldorf, Duesseldorf, Germany; 5 Klinikum Dortmund, University of Muenster, Muenster, Germany; 6 Department of Ophthalmology, and Novartis Pharma GmbH, Georg-August-University Goettingen, Nuremberg, Germany; 7 Novartis Pharma GmbH, Nuremberg, Germany; 8 Department of Ophthalmology, University Medical Center Mainz, Mainz, Germany

**Keywords:** NAMD, DME, RVO, Non-interventional study, Demographic characteristics, Anti-VEGF, Epidemiology, Ceiling effect

## Abstract

**Background:**

While randomized controlled trials (RCTs) are based on strict inclusion/exclusion criteria, non-interventional studies (NISs) might provide additional information to guide management in patients more representative to the real-world setting. The aim of this study was to compare baseline characteristics of patients receiving intravitreal treatment in the NIS OCEAN with those from published RCTs.

**Methods:**

The ongoing OCEAN study enrolled patients treated with ranibizumab for neovascular age-related macular degeneration (nAMD), diabetic macular oedema (DME) or branch/central retinal vein occlusion (B/CRVO). Baseline patient characteristics were compared by indication within the OCEAN cohort. Furthermore, the characteristics were set in reference to those of published RCTs in the same indications. Confidence intervals (CIs) were calculated and assessed for statistically significant differences as indicated by non-overlapping CIs.

**Results:**

Patient characteristics in the NIS OCEAN were evaluated for 3,614 patients with nAMD, 1,211 with DME, 204 with BRVO and 121 with CRVO. Between these groups, significant differences in mean age, gender distributions, and mean baseline VA were seen, reflecting known differences between the indications.

Compared to the patient characteristics of published RCTs (trials selected by literature search: nAMD: 13 RCTs, DME: 9, RVO: 5), the OCEAN patients’ mean age was significantly higher in every indication. The gender distributions across the trials were comparable, with only few differences between OCEAN and the RCTs. Regarding the mean baseline VA, notable differences were found in nAMD and in DME, with VA significantly higher in some RCTs and lower in others.

**Conclusions:**

The described differences underline the complementarity of NISs and RCTs. OCEAN covers a broader spectrum and more variability of patients than do RCTs. As baseline values may have impact on the treatment response (ceiling effect), there is an ongoing need for research in all patient subgroups. Country-specific assessments of patient populations can better reflect the real-world situation. NISs can deliver insights that RCTs may not, as NISs can include non-typical patients, patients with comorbidities, a broader age spectrum and patients of various disease stages.

**Trial registration:**

The NIS OCEAN was registered on www.clinicaltrials.gov (identifier: NCT02194803).

**Electronic supplementary material:**

The online version of this article (doi:10.1186/s12886-017-0401-y) contains supplementary material, which is available to authorized users.

## Background

Randomized controlled trials (RCTs) are widely considered to be the ‘gold standard’ for providing evidence for the efficacy and safety of a pharmaceutical agent. However, given the restrictions of a clinical trial setting, RCT data may not necessarily provide representative information about the real-world situation [1, 2]. Results from RCTs may be irrelevant for routine clinical practice, for example if the comparator arm does not reflect the usual standard of care or if endpoints are selected in order to maximize the statistical power rather than because of their importance to care-givers, patients and healthcare decision makers. Not only the predefined exclusion criteria, but also the selection of patients by the study investigators can contribute to the underrepresentation of, for example, elderly or low-educated patients in RCTs [3–5]. In addition, RCTs usually assess discrete relationships between treatments and outcome measures, but are not powered for observing rare events [6, 7].

Obtaining data on effectiveness and long-term safety and tolerability of therapies in routine clinical practice requires real-world observational studies.

### Strengths and limitations of non-interventional trials

Non-interventional healthcare research differs from RCTs in terms of study design, cost, relevance to clinical practice and value to payers and regulators [8]. The care-driven nature of non-interventional healthcare research, as well as the less restricted patient cohorts and longer observation timelines, help to obtain more generalizable data and a more accurate picture of the broader population and the long-term effects of a drug used within the treatment guidelines. However, observation artefacts also have to be considered, like the bias introduced by the non-randomized design and the issue of data validity (limited data collection and documentation quality). While NISs can complement RCTs due to their broader patient selection, they should also be evaluated in the context of epidemiological registry studies, which use anonymized data to obtain disease prevalences and incidences from populations that can be even more diverse than in NISs. In contrast to such epidemiological studies, NISs can provide insight into access to healthcare, i.e. analysing outcomes for those patients who actively sought medical treatment and for whom treatment was deemed necessary.

### The OCEAN study

The vast majority of diseases treated by intravitreal administration of anti-VEGF (vascular endothelial growth factor) drugs are age-dependent and known to be accompanied by comorbidities. Therefore, the risk of RCTs not assessing the representative population’s treatment needs might be even higher in these indications.

This possible discrepancy prompted the analysis herein of the baseline demographics of a large observational study in comparison with patient characteristics in the corresponding pivotal RCTs. The OCEAN study (**O**bservation of treatment patterns with Lu**CE**ntis and real life ophthalmic monitoring, including optional OCT [optical coherence tomography] in **A**pproved i**N**dications) is the largest German observational study in ophthalmology to date, focussing on the outcomes of intravitreal injections of ranibizumab (Lucentis®, Novartis Pharma) in routine clinical practice. The study is a prospective, non-interventional study (NIS) and physicians’ decisions and patients’ outcomes are documented from a purely observational perspective. Apart from ranibizumab, other anti-VEGF agents currently available are aflibercept (Eylea®, Bayer Healthcare), pegaptanib (Macugen®, Pfizer), as well as bevacizumab (Avastin®, Roche), which is used ‘off-label’ in ophthalmology.

### One NIS, three ophthalmological indications

Anti-VEGF drugs are used to address visual impairment due to neovascular age-related macular degeneration (nAMD), diabetic macular oedema (DME) and macular oedema secondary to retinal vein occlusion (RVO).

While nAMD is a major cause of impaired central vision in the elderly population [9–11], DME is reported to be the most common cause of severe visual loss in patients of working age [12]. The incidence of DME is expected to increase in future, in line with the prevalence of obesity, unhealthy diets and older age. Similarly, among the risk factors for developing RVO are older age, hypertension, cardiovascular disease and obesity [13].

In general, age is an important demographic parameter and a non-modifiable risk factor for many diseases [3–5]. At a higher age, co-morbidities may occur and behavioural patterns change due to increased physical limitations and due to changes in habits. In most countries, the demand for treatments for age-related ophthalmological indications will increase in future, in line with the demographic change [14].

Gender is another demographic characteristic that can be a non-modifiable risk factor for diseases that occur only or more frequently in one gender or the other. Male and female patients differ in how they are affected by disease. The fact that women have a higher life-expectancy than men, while they are more at risk of acquiring mental or physical disease is known as the ‘female–male health-survival paradox’ [15, 16].

In addition to the potential impact of age and gender on the occurrence and management of ocular diseases, a patient’s visual acuity (VA) at baseline can indicate limitations of the visual prognosis and can predict the potential change of VA due to a strong ceiling effect, i.e. the better a patient’s VA is at treatment start, the more improvement can be expected [17, 18].

The aim of the present analysis is to evaluate the demographic characteristics of the real-world patients from the prospective OCEAN study and to compare them to those of patients enrolled in RCTs for anti-VEGF treatments.

## Methods

The OCEAN study was initiated in December 2011 and is planned to continue until December 2016. It was approved by the responsible Ethics Committee prior to study start and written informed consent was obtained from all patients (The trial was registered on www.clinicaltrials.gov (NCT02194803).

Recruitment was completed in December 2014, with over 5,500 patients enrolled and observed in this study. The study design is prospective, non-interventional and purely observational. Prescriptions and treatment decisions are at the physicians’ discretion, intended to reflect routine clinical practice. The participating physicians are compensated for the documentation of patient data, in accordance with the official scale of physicians’ fees. Patients could be included in the study if ranibizumab treatment for one of the approved indications had been decided on prior to, and independently from, study enrolment. The treatment modalities are based on the approved German product information for Lucentis® as well as the recommendations of the scientific societies. Intravitreal injections of 0.5 mg ranibizumab are initially given on a monthly basis, followed by further treatments as needed, based on regular follow-up examinations.

For the present analysis, the demographic characteristics of the OCEAN patients at baseline (full analysis set) were compared by indication. Furthermore, they were compared with the characteristics of patients in relevant RCTs. The RCTs that were included for this comparison were selected based on a literature search of the PubMed publication database, focussing on publications reporting clinical trials of intravitreal treatments for nAMD, DME and RVO. The search results were filtered according to a set of pre-defined criteria: The RCTs had to be prospective, blinded, phase III clinical trials of intravitreal anti-VEGF treatments. The focus of the trials had to be on comparing different anti-VEGF treatments or different doses/regimens of one anti-VEGF drug. RCTs had to include predominantly Caucasian subjects (in line with the German OCEAN study) and the baseline demographics of the respective study had to be available in English (abstract and full text). Only RCTs with more than 60 patients in total were included in the analysis, in order to focus on larger, more representative multi-centre studies with less selection bias.

The following demographic parameters were evaluated for OCEAN as well as for each RCT, by treatment arm, where available in the original publications: age, gender, body mass index (BMI), time since diagnosis of study indication and baseline VA (in Early Treatment Diabetic Retinopathy Study [ETDRS] letters). It should be noted that the exact method of obtaining baseline VA was not always reported in the respective publication; therefore, the comparisons of VA results across studies have to be treated with caution. The results are presented by indication. For nAMD, information on the patients’ medical history (hypertension, myocardial infarction, stroke/apoplexy, transient ischemic attack) was recorded. For DME, diabetes type, mean glycated haemoglobin (HbA1c) level and mean time since diagnosis of diabetes were additionally recorded; for RVO, the type of RVO (CRVO or BRVO) was differentiated. The comparison of the demographic characteristics was purely descriptive. For all parameters and all trials, the 95% confidence intervals (CIs) were calculated, if sufficient details (N and mean ± standard deviation [SD] or percentage) were available. The CIs for the OCEAN populations (by indication) were compared with each other and with the CIs of the respective RCTs. All results for which the CIs showed no overlap were considered statistically significantly different for the purpose of the present analysis.

## Results

### Baseline characteristics of the OCEAN study population

A total of 5,606 patients (full analysis set) were included in the NIS OCEAN at the time of its fifth interim analysis, and after recruitment was completed. Of these, 3,614 patients were enrolled for treatment of nAMD, 1,211 patients were included for treatment of DME and 741 patients were treated for RVO, of which 204 patients were documented with a sub-diagnosis of BRVO and 121 with CRVO (Table 1). The baseline characteristics of the OCEAN populations are described in the following (further details and CIs provided in Additional file 1: Table S1).

**Table 1:** Summary of patient demographics for the OCEAN population, by indication

Primary indication	*N*	Age mean ± SD (years)	Gender, *n* (%)		BMI mean ± SD (kg/m^2^)	Time since diagnosis of primary indication mean ± SD (years)^a^	Baseline VA
			male	female			ETDRS letters analogue (mean ± SD)
Overall^b^	5606	74.6 ± 10.3	2451 (43.72)	3136 (55.94)	27.3 ± 4.5	0.56 ± 1.38	53.9 ± 20.6
nAMD^c^	3614	77.9 ± 8.2	1393 (38.5)	2210 (61.2)	26.6 ± 4.0	0.53 ± 1.28	52.0 ± 21.3
DME^d^	1211	67.6 ± 10.9	698 (57.6)	507 (41.9)	29.3 ± 5.2	0.68 ± 1.63	60.6 ± 15.5
RVO^e^	741	71.0 ± 11.0	350 (47.23)	389 (52.50)	27.1 ± 4.3	0.49 ± 1.30	52.0 ± 22.7
BRVO^f^	204	71.2 ± 10.0	85 (41.7)	119 (58.3)	27.1 ± 4.3	0.53 ± 1.37	55.9 ± 20.9
CRVO^g^	121	70.3 ± 11.5	57 (47.1)	64 (52.9)	26.8 ± 4.4	0.32 ± 0.54	43.7 ± 25.0

Further details for the OCEAN populations are provided in Additional file 1: Table S1, Additional file 3: Table S3, Additional file 4: Table S4, Additional file 5: Table S5, Additional file 6: Table S6, Additional file 7: Table S7

^a^ Time since diagnosis of primary indication until first injection in OCEAN study

^b^ Overall: Missing values: age: 25 patients; gender: 19; BMI: 413; time since diagnosis: 281; baseline VA: 47

^c^ nAMD: Missing values: age: 14 patients; gender: 11; BMI: 230; time since diagnosis: 199; baseline VA: 29

^d^ DME: Missing values: age: 9 patients; gender: 6; BMI: 87; time since diagnosis: 65; baseline VA: 8

^e^ RVO: Missing values: type of RVO (B/C): 416 patients; age: 2; gender: 2; BMI: 56; time since diagnosis: 17; baseline VA: 10

^f^ BRVO: Missing values: age and gender: 0 patients; BMI: 8; time since diagnosis: 3; baseline VA: 1

^g^ CRVO: Missing values: age and gender: 0 patients; BMI: 2; time since diagnosis: 3; baseline VA: 3

Abbreviations: BMI: body mass index; BRVO: branch retinal vein occlusion; CRVO: central retinal vein occlusion; DME: diabetic macular oedema; ETDRS: Early Treatment Diabetic Retinopathy Study; N: total number of patients; *n*: number of patients; nAMD: neovascular age-related macular degeneration; RVO: retinal vein occlusion; SD: standard deviation; VA: visual acuity

The OCEAN population’s overall mean age (±SD) at baseline was 74.6 ± 10.3 years. The mean age of the nAMD patients (77.9 ± 8.2 years; 95% CI [77.6; 78.2] years) was significantly higher than that of the patients in the other indications (95% CIs lower for other indications [upper 95% CI limits <77.6 years], non-overlapping). In contrast, the DME patients (67.6 ± 10.9 years; 95% CI [67.0; 68.2] years) were significantly younger than all others (95% CIs higher for other indications [lower 95% CI limits >68.2 years], non-overlapping).

Overall, the OCEAN study included more female patients (3,136 patients, 55.94%) than male patients (2,451 patients, 43.72%). This difference between the genders was statistically significant in the nAMD and BRVO populations (non-overlapping 95% CIs for percentages of females and males per indication). In DME, the situation was reversed, with significantly more male than female patients (non-overlapping 95% CIs). When comparing the different indications, none of the indications differed significantly from the others, although the proportion of males was slightly lower in nAMD compared to the others, and slightly higher in DME compared to the others.

The mean BMI was significantly higher in the DME patients (29.3 ± 5.2 kg/m^2^; 95% CI [29.01; 29.59] kg/m^2^) than in all other indications (95% CIs lower for other indications [upper 95% CI limits <29.01 kg/m^2^], non-overlapping).

The time since the diagnosis of the OCEAN patients’ primary indication until their first treatment during the OCEAN observation period ranged between 0.3 and 0.7 years, albeit with comparably high SDs (and mostly overlapping 95% CIs).

At OCEAN study baseline, the mean VA (± SD) was 53.9 ± 20.6 letters for the overall population. In DME, the mean baseline VA (60.6 ± 15.5 letters; 95% CI [59.7; 61.5] letters) was significantly higher than that of the patients in the other indications (95% CIs lower for other indications [upper 95% CI limits <59.7 letters], non-overlapping). The mean baseline VA was significantly lower in CRVO (43.7 ± 25.0 letters; 95% CI [39.2; 48.2] letters), compared to the other indications (95% CIs higher for other indications [lower 95% CI limits >48.2 letters], non-overlapping).

Further indication-specific baseline characteristics of the OCEAN population are described below.

### Comparison of real-world OCEAN population with randomized controlled trials

**Fig. 1:**
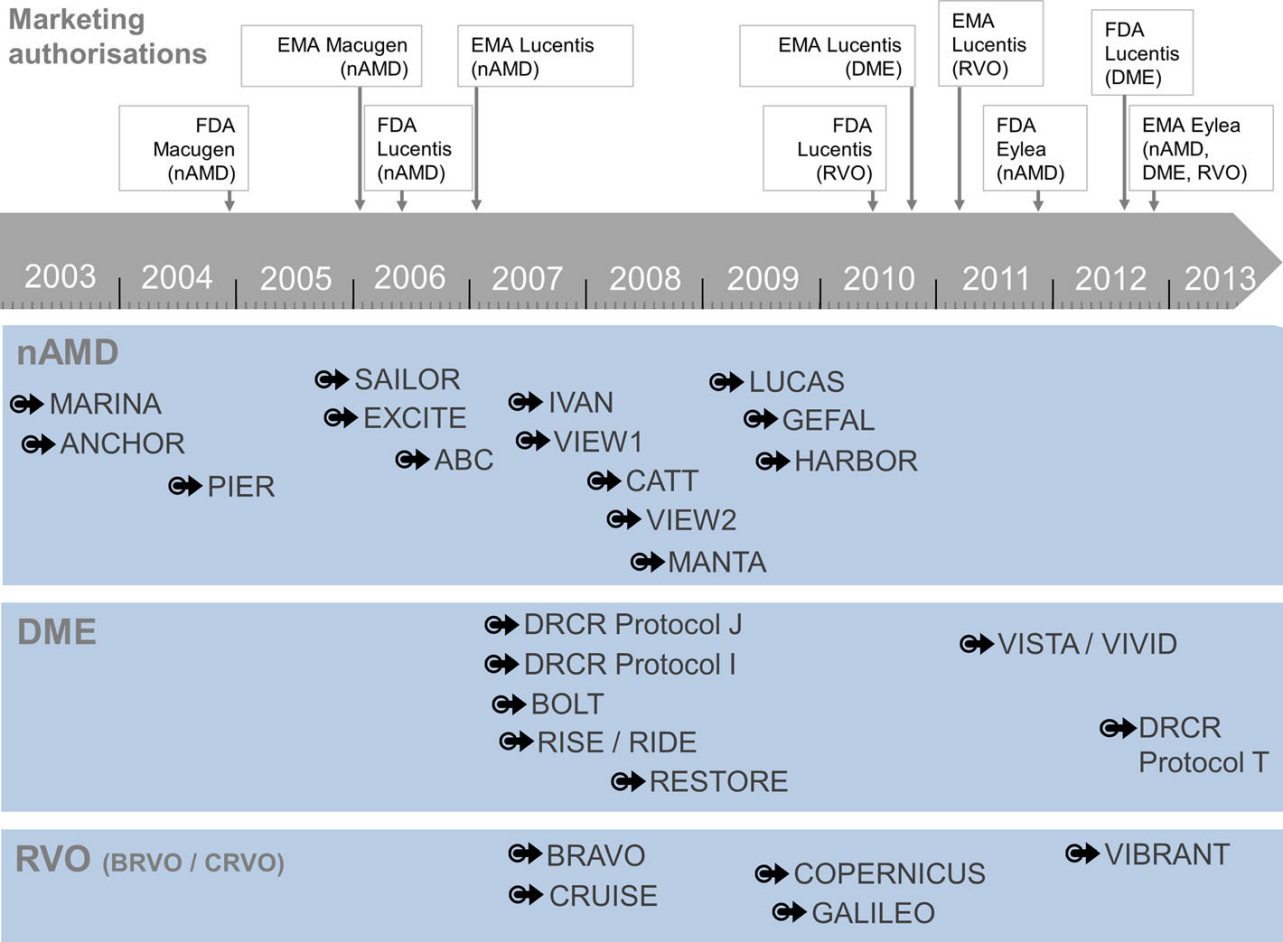
Start dates of randomized controlled trials by indication. *Legend*: RCT start dates shown in relation to EMA and FDA ophthalmological marketing authorisation dates for Eylea®, Lucentis® and Macugen®. For sources of details and study periods for the RCTs see Additional file 2: Table S2. Abbreviations: BRVO: branch retinal vein occlusion; CRVO: central retinal vein occlusion; DME: diabetic macular oedema; EMA: European Medicines Agency; FDA: US Food and Drug Administration; nAMD: neovascular age-related macular degeneration; RCT: randomized controlled trial; RVO: retinal vein occlusion

A literature review using PubMed (www.ncbi.nlm.nih.gov/pubmed, restricted to clinical trials only, performed in October 2015) yielded 235 results for the indication nAMD (search term “intravitreal AND neovascular”), 240 results for DME (search term “intravitreal AND diabetic AND edema”) and 147 results for RVO (search term “intravitreal AND vein AND occlusion”). The publications were screened for trials fulfilling the pre-defined criteria. The final set of RCTs for comparison with OCEAN included thirteen nAMD trials, nine DME trials and five RVO trials. Details for these trials are presented in Additional file 2: Table S2 and an overview of the time periods of the RCTs in relation to the marketing authorization dates of the approved anti-VEGF agents is shown in Fig. 1.

The identified RCTs were compared with the OCEAN baseline data. The patient populations of the RCTs were presented by treatment group in the source publications, with two to six treatment groups/arms per study, each with varying baseline demographics. As OCEAN is an open-label observational study, the OCEAN population is shown in total and not sub-divided into any treatment subgroups.

### Comparison of OCEAN and RCTs for nAMD

**Fig. 2:**
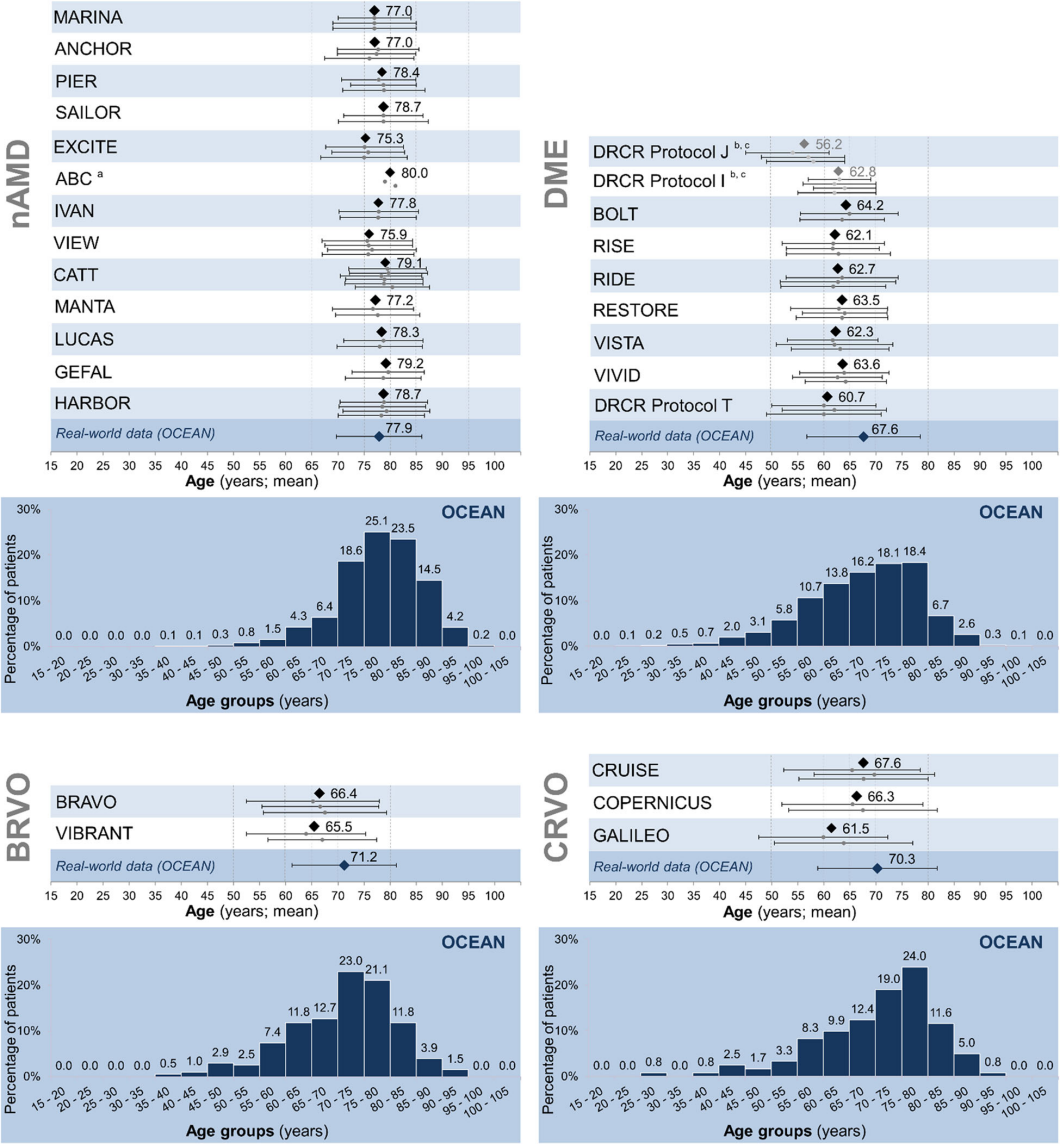
Baseline age distribution in OCEAN and patient age for OCEAN and RCTs, by indication. *Legend*: Age distribution in years by age group for OCEAN and mean age in years (with SD) for OCEAN and RCTs. Mean age is given overall per study (black square) and as mean with SD for each individual treatment group (grey circles with error bars). If the mean was not available, the median is shown (grey squares) with 25^th^/75^th^ percentiles. The data for the individual treatment groups of each RCT are shown in the same order from top to bottom as the treatment groups are presented in Tables 2, 3, 4. ^a^no SD available ^b^Data provided as median (25^th^, 75^th^ percentile). ^c^Data provided for number of eyes, not number of patients. Abbreviations: BRVO: branch retinal vein occlusion; CRVO: central retinal vein occlusion; DME: diabetic macular oedema; nAMD: neovascular age-related macular degeneration; RCT: randomized controlled trial; SD: standard deviation

The baseline parameters of the 3,614 nAMD patients in OCEAN were compared with those from the selected RCTs in nAMD (for details see Table 2 and Additional file 3: Table S3 and Additional file 4: Table S4 [CIs]): The mean age (± SD) of the nAMD patients in OCEAN was 77.9 ± 8.2 years (95% CI [77.6; 78.2] years), in line with the mean age across the RCTs (ranging from 75.0 to 81 years) (Fig. 2). At the individual study level, the OCEAN patients tended to be younger than those included in SAILOR, CATT, GEFAL and HARBOR, and this difference was statistically significant for at least one of the treatment arms in each of these RCTs (non-overlapping 95% CIs). In contrast, the OCEAN patients were significantly older than the patients in all treatment arms of the RCTs EXCITE and VIEW (non-overlapping 95% CIs). A notable difference between OCEAN and the RCTS was also observed in the age range, which was broader in OCEAN (36 to 101 years) than in the other studies where this information was available (minimum age 50 years [usually inclusion criterion in RCTs], up to 101 years).

**Table 2:** Patient demographics for OCEAN and selected randomized controlled trials in the indication neovascular age-related macular degeneration

Study	Treatment group	*N*	Age mean ± SD (years)	Age range (years)	Gender, *n* (%)		Time since diagnosis of nAMD mean ± SD (years)	Patients with medical history of				Baseline VA
					male	female		hyper-tension (n [%])	myocardial infarction (*n* [%])	stroke or apoplexy (*n* [%])	transient ischemic attack (*n* [%])	ETDRS letters analogue (mean ± SD)^a^
OCEAN^b^	Ranibizumab 0.5 mg	3614	77.9 ± 8.2	36-101	1393 (38.5)	2210 (61.2)	0.53 ± 1.28 ^c,d^	804 (22.3)	198 (5.5)	146 (4.0)	2 (0.1)	52.0 ± 21.3
MARINA [30]	Sham injections q4	238	77 ± 7	56-94	79 (33.2)	159 (66.8)	n. a.	n. a.	n. a.	n. a.	n. a.	53.6 ± 14.1
	Ranibizumab 0.3 mg q4	238	77 ± 8	52-95	85 (35.7)	153 (64.3)	n. a.	n. a.	n. a.	n. a.	n. a.	53.1 ± 12.9
	Ranibizumab 0.5 mg q4	240	77 ± 8	52-93	88 (36.7)	152 (63.3)	n. a.	n. a.	n. a.	n. a.	n. a.	53.7 ± 12.8
ANCHOR [31]^e^	Verteporfin + sham injections + laser	143	77.7 ± 7.8	53-95	64 (44.8)	79 (55.2)	n. a.	n. a.	n. a.	n. a.	n. a.	45.5 ± 13.1
	Ranibizumab 0.3 mg + sham verteporfin + laser	140	77.4 ± 7.5	54-97	73 (52.1)	67 (47.9)	n. a.	n. a.	n. a.	n. a.	n. a.	47.0 ± 13.1
	Ranibizumab 0.5 mg + sham verteporfin + laser	140	76.0 ± 8.6	54-93	75 (53.6)	65 (46.4)	n. a.	n. a.	n. a.	n. a.	n. a.	47.1 ± 13.2
PIER [32]^f^	Sham	63	77.8 ± 7.1	59-92	20 (31.7)	43 (68.3)	0.3 ± 0.5	n. a.	n. a.	n. a.	n. a.	55.1 ± 13.9
	Ranibizumab 0.3 mg	60	78.7 ± 6.3	60-93	26 (43.3)	34 (56.7)	0.7 ± 1.6	n. a.	n. a.	n. a.	n. a.	55.8 ± 12.2
	Ranibizumab 0.5 mg	61	78.8 ± 7.9	54-94	28 (45.9)	33 (54.1)	0.7 ± 1.2	n. a.	n. a.	n. a.	n. a.	53.7 ± 15.5
SAILOR [33]^g^	Cohort 1 arm 1: Ranibizumab 0.3 mg	1169	78.7 ± 7.6	51-97	n. a.	59.9 ^h^	0.3 ± 1.4 ^i^ 1.4 ± 2.0 ^k^	n. a.	n. a.	n. a.	n. a.	55.0 ± 12.5 ^i^53.8 ± 13.8^k^
	Cohort 1 arm 2: Ranibizumab 0.5 mg	1209	78.7 ± 8.6	52-101	n. a.	58.1 ^h^	0.3 ± 0.7 ^i^ 1.3 ± 1.7 ^k^	n. a.	n. a.	n. a.	n. a.	48.9 ± 13.8 ^i^50.0 ± 14.3^k^
EXCITE [34]	Ranibizumab 0.3 mg q16	120	75.1 ± 7.45	n. a.	50 (41.7)	70 (58.3)	0.57 ± 1.42	n. a.	n. a.	n. a.	n. a.	55.8 ± 11.81
	Ranibizumab 0.5 mg q16	118	75.8 ± 6.96	n. a.	45 (38.1)	73 (61.9)	0.52 ± 1.14	n. a.	n. a.	n. a.	n. a.	57.7 ± 13.06
	Ranibizumab 0.3 mg q4	115	75 ± 8.26	n. a.	49 (42.6)	66 (57.4)	0.56 ± 2.18	n. a.	n. a.	n. a.	n. a.	56.5 ± 12.19
ABC [35]	Bevacizumab 1.25 mg, q6	65	79 ^m^	n. a.	26 (40)	39 (60)	n. a.	n. a.	n. a.	n. a.	n. a.	50 (43–61) ^n^
	Standard therapy^o^	66	81 ^m^	n. a.	25 (38)	41 (62)	n. a.	n. a.	n. a.	n. a.	n. a.	53 (47–60) ^n^
IVAN [36]	Ranibizumab 0.5 mg	314	77.8 ± 7.6	n. a.	129 (41)	n. a.	n. a.	n. a.	24 (8)	7 (2)	20 (6) ^p^	61.8 ± 15.0
	Bevacizumab 1.25 mg	296	77.7 ± 7.3	n. a.	115 (39)	n. a.	n. a.	n. a.	22 (7)	7 (2) ^q^	9 (3) ^p^	61.1 ± 15.5
VIEW [37] (VIEW1 + VIEW2 pooled)	Ranibizumab 0.5 mg q4	595	75.6 ± 8.7	n. a.	n. a.	341 (57.3)	n. a.	n. a.	n. a.	n. a.	n. a.	53.9 ± 13.4
	Aflibercept 2 mg q4	613	75.9 ± 8.4	n. a.	n. a.	370 (60.4)	n. a.	n. a.	n. a.	n. a.	n. a.	54.0 ± 13.6
	Aflibercept 0.5 mg q4	597	76.5 ± 8.5	n. a.	n. a.	314 (52.6)	n. a.	n. a.	n. a.	n. a.	n. a.	53.6 ± 13.8
	Aflibercept 2 mg q8	607	75.8 ± 8.8	n. a.	n. a.	353 (58.2)	n. a.	n. a.	n. a.	n. a.	n. a.	53.6 ± 13.5
CATT [38]	Ranibizumab 0.5 mg q4	146	79.5 ± 7.4	n. a.	56 (38.4)	90 (61.6)	n. a.	102 (69.9)	15 (10.3)	6 (4.1)	8 (5.5)	59.9 ± 14.2
	Bevacizumab 1.25 mg q4	135	79.7 ± 7.5	n. a.	53 (39.3)	82 (60.7)	n. a.	93 (68.9)	16 (11.9)	7 (5.2)	12 (8.9)	60.2 ± 13.6
	Ranibizumab 0.5 mg PRN	287	78.3 ± 7.8	n. a.	108 (37.6)	179 (62.4)	n. a.	195 (67.9)	28 (9.8)	22 (7.7)	11 (3.8)	61.6 ± 13.1
	Bevacizumab 1.25 mg PRN	270	78.9 ± 7.4	n. a.	104 (38.5)	166 (61.5)	n. a.	196 (72.6)	33 (12.2)	16 (5.9)	17 (6.3)	60.6 ± 13.0
	Ranibizumab 0.5 mg year 1 q4, year 2 PRN	138	78.8 ± 7.5	n. a.	56 (40.6)	82 (59.4)	n. a.	97 (70.3)	17 (12.3)	7 (5.1)	4 (2.9)	60.9 ± 14.3
	Bevacizumab 1.25 mg year 1 q4, year 2 PRN	131	80.4 ± 7.1	n. a.	45 (34.4)	86 (65.6)	n. a.	84 (64.1)	19 (14.5)	9 (6.9)	11 (8.4)	60.4 ± 12.4
MANTA [39]	Bevacizumab 1.25 mg	154	76.7 ± 7.8	n. a.	56 ^r^	98 ^r^	n. a.	n. a.	n. a.	n. a.	n. a.	57.0 ± 13.0
	Ranibizumab 0.5 mg	163	77.6 ± 8.1	n. a.	59 ^r^	104 ^r^	n. a.	n. a.	n. a.	n. a.	n. a.	56.4 ± 13.5
LUCAS [40]	Bevacizumab 1.25 mg	213	78.7 ± 7.6	n. a.	62 (29.1)	151 (70.9)	n. a.	n. a.	12 (5.6)	11 (5.2) ^s^	11 (5.2)	60 ± 14
	Ranibizumab 0.5 mg	218	78.0 ± 8.2	n. a.	78 (35.8)	140 (64.2)	n. a.	n. a.	26 (11.9)	11 (5.0) ^s^	12 (5.5)	62 ± 13
GEFAL [41]	Bevacizumab 1.25 mg	191	79.62 ± 6.90	55.8-97.6	72 (37.7)	119 (62.3)	n. a.	119 (62.3)	10 (5.2)	7 (3.7)	1 (0.5)	54.62 ± 14.07
	Ranibizumab 0.5 mg	183	78.68 ± 7.27	52.1-95.6	54 (29.5)	129 (70.5)	n. a.	94 (51.4)	3 (1.6)	3 (1.6)	0 (0.0)	55.78 ± 13.99
HARBOR [42]	Ranibizumab 0.5 mg q4	275	78.8 ± 8.4	53-97	113 (41.1)	162 (58.9)	n. a.	n. a.	n. a.	n. a.	n. a.	54.2 ± 13.3
	Ranibizumab 0.5 mg PRN	275	78.5 ± 8.3	53-97	112 (40.7)	163 (59.3)	n. a.	n. a.	n. a.	n. a.	n. a.	54.5 ± 11.7
	Ranibizumab 2.0 mg q4	274	79.3 ± 8.3	50-96	104 (38.0)	170 (62.0)	n. a.	n. a.	n. a.	n. a.	n. a.	53.5 ± 13.1
	Ranibizumab 2.0 mg PRN	273	78.3 ± 8.3	54-98	117 (42.9)	156 (57.1)	n. a.	n. a.	n. a.	n. a.	n. a.	53.5 ± 13.2

^a^ Method of measuring baseline VA not always explained in sources. Direct comparisons of VA results may not be reliable

^b^ Missing values in OCEAN: age: 14 patients; gender: 11; time since diagnosis: 199; medical history: 27; baseline VA: 29

^c^ Time since diagnosis until first injection in OCEAN

^d^ Results converted to years (originally in days)

^e^ Missing values: VA in 0.5 mg ranibizumab group: 1 patient

^f^ Missing values: Time since diagnosis: sham: 1 patient, 0.3 mg ranibizumab: 1 patient

^g^ Only cohort 1 of the SAILOR trial included here, as this was blinded. Cohort 2 received open-label treatment

^h^ Data in percent only

^i^ Data for the treatment-naïve patients in this treatment arm

^k^ Data for the previously treated patients in this treatment arm

^m^ No SD available for the mean age

^n^ Data given as median (25^th^, 75^th^ percentile)

^o^ Standard therapy: pegaptanib: 38 patients, verteporfin: 16, sham: 12

^p^ Missing values: ranibizumab: 20 patients, bevacizumab: 14

^q^ Missing values: bevacizumab: 1 patient

^r^ Only number of patients; no percentage available

^s^ Stroke defined as “cerebrovascular infarction” in this study

Abbreviations: *ETDRS*: Early Treatment Diabetic Retinopathy Study; *N*: total number of patients; n: number of patients; n. a.: data not available in source; *nAMD*: neovascular age-related macular degeneration; *PRN*: pro re nata (as needed); q4: every 4 weeks; q6: every 6 weeks; q8: every 4 weeks for 3 months followed by dosing every 8 weeks; q16: every 4 months (quarterly); *SD*: standard deviation; *VA*: visual acuity

**Fig. 3:**
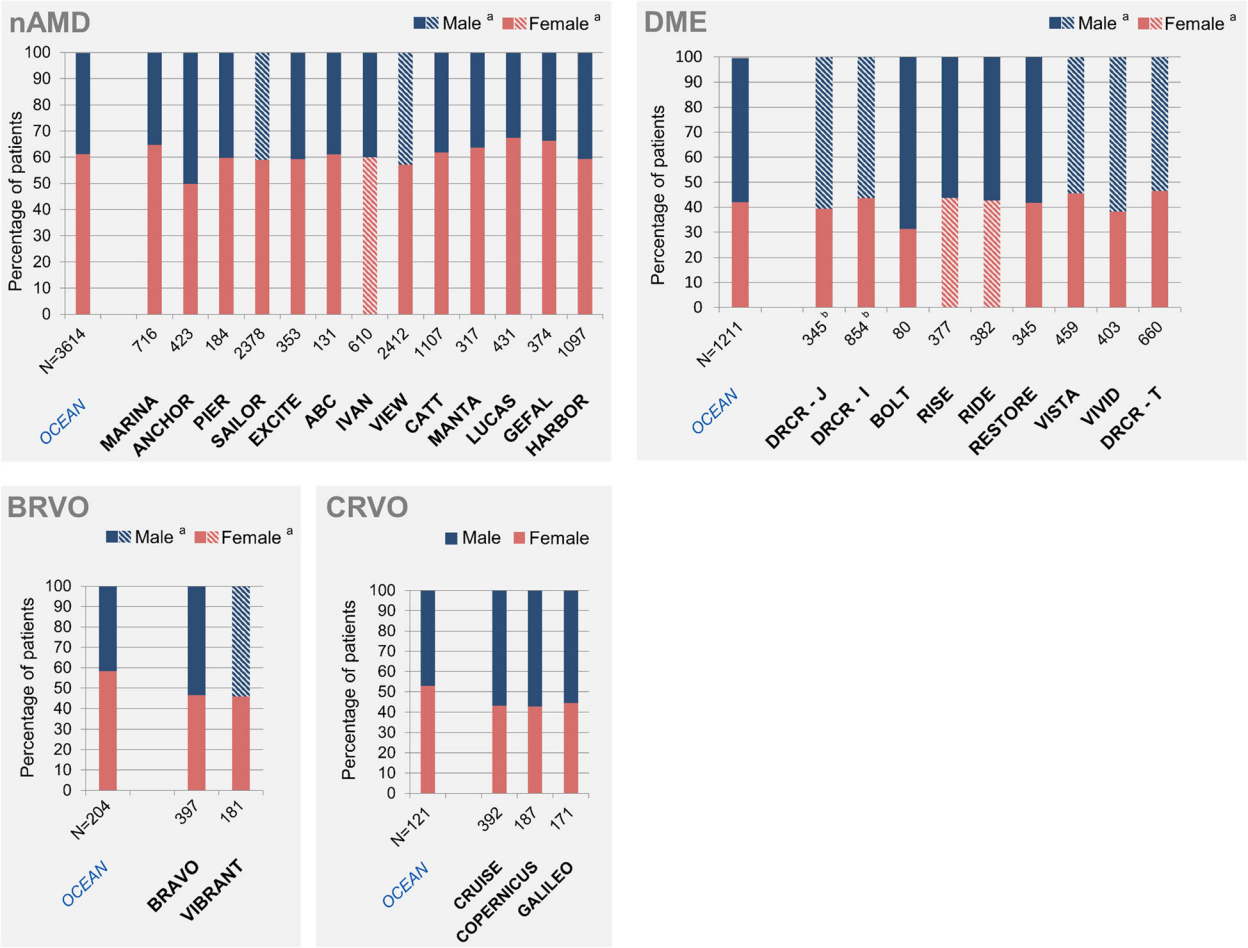
Gender distribution in OCEAN study and in selected RCTs, by indication. *Legend:* Proportion of female patients (red) and male patients (blue) at baseline. ^a^Hatched columns: Gender group not explicitly provided in source data; data for respective other gender used for calculation of percentage (may include missing/unavailable data). ^b^Data provided for number of eyes, not number of patients. Abbreviations: BRVO: branch retinal vein occlusion; CRVO: central retinal vein occlusion; DME: diabetic macular oedema; *N*: number of patients; nAMD: neovascular age-related macular degeneration; RCT: randomized controlled trial

More female than male patients were treated in OCEAN (2,210 females, 61.2%, 95% CI [59.5; 62.7] %; 1,393 males, 38.5%, 95% CI [36.9; 40.1] %), and the situation was comparable in most of the RCTs (range: 52.6% to 70.9% females; Fig. 3). The most notable exception was ANCHOR, where more male than female patients were included in two of three treatment arms (significant difference to OCEAN, with non-overlapping 95% CIs).

**Fig. 4:**
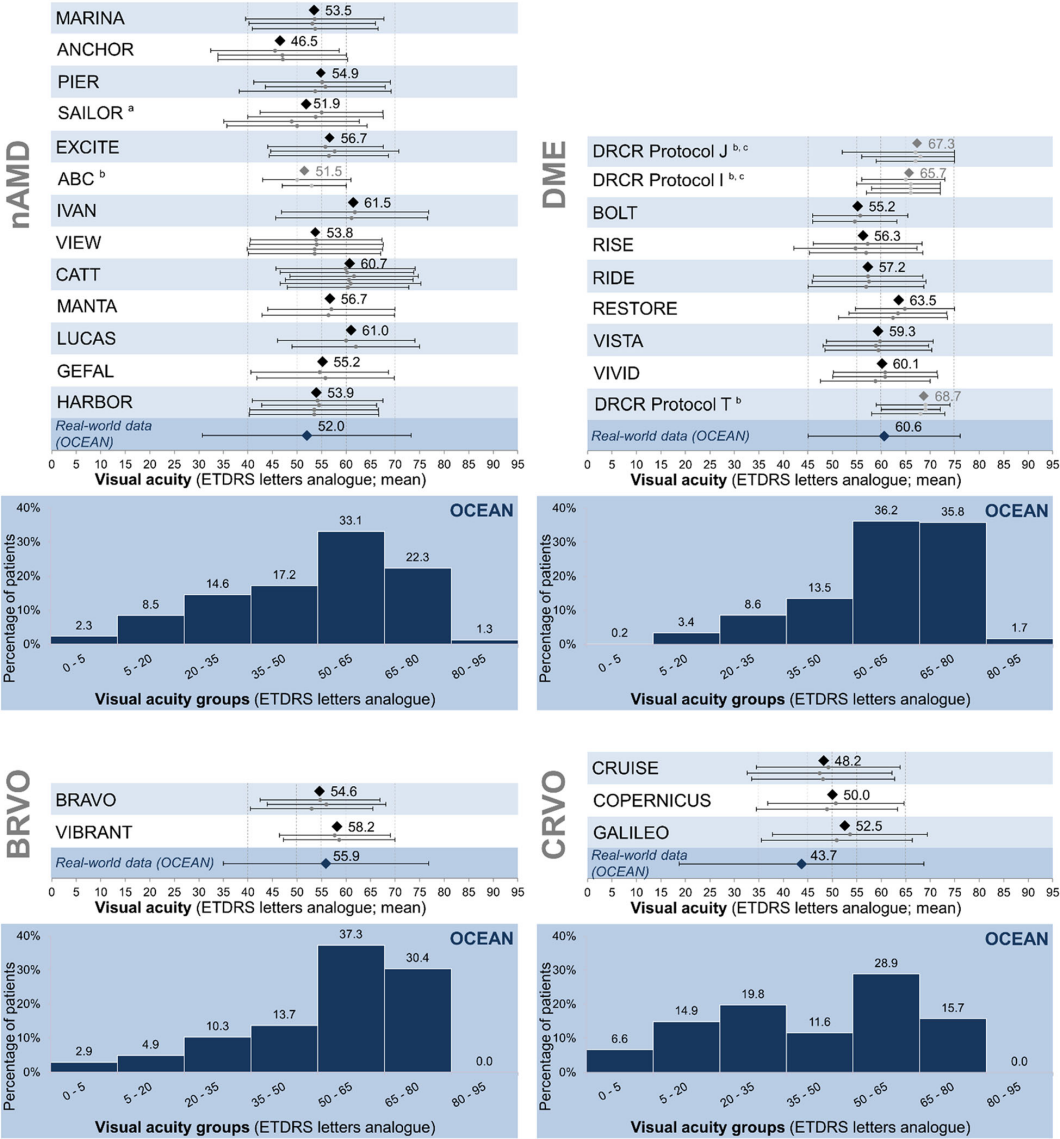
Baseline visual acuity distribution in OCEAN and visual acuity for OCEAN and RCTs, by indication. *Legend:* Visual acuity distribution in ETDRS letter analogues by group for OCEAN and mean visual acuity (in ETDRS letter analogues with SD) for OCEAN and RCTs. Mean visual acuity is given overall per study (black square) and as mean with SD for each individual treatment group (grey circles with error bars). If the mean was not available, the median is shown (grey square) with 25^th^/75^th^ percentiles. The data for the individual treatment groups of each RCT are shown in the same order from top to bottom as the treatment groups are presented in Tables 2, 3, 4. ^a^The visual acuity results for SAILOR are provided for four treatment groups. ^b^Data provided as median (25^th^, 75^th^ percentile). ^c^ Data provided for number of eyes, not number of patients. Abbreviations: BRVO: branch retinal vein occlusion; CRVO: central retinal vein occlusion; DME: diabetic macular oedema; ETDRS: Early Treatment Diabetic Retinopathy Study; nAMD: neovascular age-related macular degeneration; RCT: randomized controlled trial; SD: standard deviation

The patients’ mean VA (ETDRS letters ± SD) at baseline was 52.0 ± 21.3 letters in OCEAN (95% CI [51.3; 52.7] letters), while it ranged from 45.5 to 62 letters in the RCTs (Fig. 4). The baseline VA in OCEAN was significantly higher than in ANCHOR and in two of four treatment arms in SAILOR, while it was significantly lower than in EXCITE, IVAN, CATT, MANTA and LUCAS, and for some of the treatment arms in SAILOR, VIEW, GEFAL and HARBOR (significant differences to OCEAN with non-overlapping 95% CIs).

Data on patients’ BMI were only available for the OCEAN study in nAMD (mean ± SD: 26.6 ± 4.0 kg/m^2^), therefore a comparison with the RCTs is not possible.

In OCEAN, the mean time since the initial diagnosis of nAMD was half a year, albeit with a high SD (1.3 years). This result was similar or slightly higher than in the RCTs for which this information was available: PIER (0.3 to 0.7 years), SAILOR (0.3 to 1.4 years) and EXCITE (0.52 to 0.57 years). Due to the high SDs compared to the means for the time since diagnosis, the data do not allow a reliable evaluation.

The medical history of the nAMD patients, recorded in OCEAN and in some of the RCTs, included prior hypertension, myocardial infarction, stroke/apoplexy and transient ischemic attack. In OCEAN, prior hypertension was documented for 804 nAMD patients (22.3%; 95% CI [20.9; 23.6] %), a statistically significantly lower incidence rate than in the RCTs for which this information was available, CATT and GEFAL (range: 51.4% to 72.6%; 95% CIs non-overlapping with OCEAN). The percentage of OCEAN patients with prior myocardial infarction was 5.5% (198 patients; 95% CI [4.8; 6.3] %) and higher than this in most of the RCTs (range from 1.6% to 14.5%). This difference compared to OCEAN reached statistical significance for most treatment arms of CATT and the ranibizumab arm of LUCAS (non-overlapping 95% CIs). Prior stroke/apoplexy was documented for 146 patients (4.0%, 95% CI [3.4; 4.7] %) in OCEAN and at a similar or only slightly higher level in the RCTs. The incidence of prior transient ischemic attack was reported as very low in OCEAN (2 patients, 0.1%, 95% CI [0.0; 0.2] %) and was statistically significantly higher than this in the RCTs IVAN, CATT and LUCAS (2.9% to 8.9% of patients, non-overlapping 95% CIs) (Table. 2).

### Comparison of OCEAN and RCTs for DME

When comparing the baseline demographic parameters of the 1,211 DME patients in OCEAN with those included in the selected RCTs for DME, a number of differences were observed (for details see Table 3 and Additional file 5: Table S5 and Additional file 6: Table S6 [CIs]): The OCEAN patients were older (mean ± SD: 67.6 ± 10.9 years, 95% CI [67.0; 68.2] years) than those in the RCTs (means ranging from 60 to 64.9 years; Fig. 2). This difference was statistically significant for almost all RCTs where a mean age was available (RISE, RIDE, RESTORE, VISTA, VIVID, DRCR.net Protocol T, one treatment arm in BOLT; all with 95% CIs non-overlapping with OCEAN).

**Table 3:** Patient demographics for OCEAN and selected randomized controlled trials in the indication diabetic macular oedema

Study	Treatment group	*N*	Age mean ± SD (years)	Age range (years)	Gender, *n* (%)		BMI mean ± SD (kg/m^2^)	Diabetes type, *n* (%)		HbA_1c_mean ± SD (%)	Time since diagnosis of diabetes mean ± SD (years)	Time since diagnosis of DME mean ± SD (years)	Time since diagnosis of DME median [range] (years)	Baseline VA ETDRS letters analogue (mean ± SD) ^a^
					male	female		Type 1	Type 2					
OCEAN ^b^	Ranibizumab 0.5 mg	1211	67.6 ± 10.9	23-100	698 (57.6)	507 (41.9)	29.3 ± 5.2	116 (9.6)	936 (77.3)	7.5 ± 1.3	n. a.	0.68 ± 1.63 ^c, d^	0.16 [0–16] ^d^	60.6 ± 15.5
DRCR.net Protocol J [43] ^e^	Sham injection + laser	123	54 (45,61) ^f^	n. a.	n. a.	44 (36)	n. a.	20 (16)	101 (82)	7.9 (7.0, 9.6) ^f^	15 (8, 21) ^f^	n. a.	n. a.	67 (52, 75) ^f^
	Ranibizumab + laser	113	57 (48,64) ^f^	n. a.	n. a.	48 (42)	n. a.	13 (12)	93 (82)	8.1 (7.1, 9.9) ^f^	15 (10, 21) ^f^	n. a.	n. a.	68 (56, 75) ^f^
	Triamcinolone + laser	109	58 (49,64) ^f^	n. a.	n. a.	44 (40)	n. a.	12 (11)	95 (87)	8.1 (7.0, 9.7) ^f^	15 (10, 19) ^f^	n. a.	n. a.	67 (59, 75) ^f^
DRCR.net Protocol I [44] ^g^	Sham injection + prompt laser	293	63 (57,69) ^f^	n. a.	n. a.	123 (42)	n. a.	25 (9)	260 (89)	7.3 (6.6, 8.3) ^f^	16 (9, 22) ^f^	n. a.	n. a.	65 (56, 73) ^f^
	Ranibizumab + prompt laser	187	62 (56,70) ^f^	n. a.	n. a.	85 (45)	n. a.	11 (6)	172 (92)	7.3 (6.6, 8.4) ^f^	18 (12, 24) ^f^	n. a.	n. a.	66 (55, 72) ^f^
	Ranibizumab + deferred laser	188	64 (58,70) ^f^	n. a.	n. a.	78 (41)	n. a.	15 (8)	170 (90)	7.5 (6.7, 8.4) ^f^	17 (11, 22) ^f^	n. a.	n. a.	66 (58, 72) ^f^
	Triamcinolone + prompt laser	186	62 (55,70) ^f^	n. a.	n. a.	86 (46)	n. a.	14 (8)	166 (89)	7.4 (6.5, 8.6) ^f^	17 (11, 24) ^f^	n. a.	n. a.	66 (57, 72) ^f^
BOLT [45]	Bevacizumab	42	64.9 ± 9.4	n. a.	30 ^h^	12 ^h^	n. a.	4	38	7.6 ± 1.4	13.5 ^i^	n. a.	2 [1.5-4] ^i^	55.7 ± 9.7
	Laser	38	63.5 ± 8.1	n. a.	25 ^h^	13 ^h^	n. a.	4	34	7.5 ± 1.2	14.8 ^i^	n. a.	3 [2–4.6] ^i^	54.6 ± 8.6
RISE [46] ^k^	Sham injections	127	61.8 ± 9.8	39-85	74 (58.3)	n. a.	31.4 ± 7.1	n. a.	n. a.	7.7 ± 1.5	14.5 ± 9.9	2.3 ± 3.0 ^m^	n. a.	57.2 ± 11.11
	Ranibizumab 0.3 mg	125	61.7 ± 8.9	38-82	73 (58.4)	n. a.	32.3 ± 6.8	n. a.	n. a.	7.7 ± 1.5	15.9 ± 9.9	2.1 ± 2.2 ^m^	n. a.	54.7 ± 12.6
	Ranibizumab 0.5 mg	125	62.8 ± 10.0	21-87	65 (52.0)	n. a.	32.9 ± 8.5	n. a.	n. a.	7.7 ± 1.4	16.3 ± 8.5	2.1 ± 2.1 ^m^	n. a.	56.9 ± 11.6
RIDE [46] ^n^	Sham injections	130	63.5 ± 10.8	22-91	66 (50.8)	n. a.	32.3 ± 8.9	n. a.	n. a.	7.6 ± 1.4	16.6 ± 10.6	2.4 ± 3.2 ^m^	n. a.	57.3 ± 11.2
	Ranibizumab 0.3 mg	125	62.7 ± 11.1	24-88	73 (58.4)	n. a.	32.3 ± 8.6	n. a.	n. a.	7.6 ± 1.3	16.0 ± 9.8	1.6 ± 2.0 ^m^	n. a.	57.5 ± 11.6
	Ranibizumab 0.5 mg	127	61.8 ± 10.1	29-84	80 (63.0)	n. a.	31.3 ± 7.2	n. a.	n. a.	7.6 ± 1.5	15.3 ± 10.1	1.9 ± 2.4 ^m^	n. a.	56.9 ± 11.8
RESTORE [47]	Ranibizumab 0.5 mg + sham laser	116	62.9 ± 9.29	n. a.	73 (62.9)	43 (37.1)	n. a.	13 (11.2)	103 (88.8)	n. a. ^o^	15.23 ± 9.91	1.80 ± 1.98	n. a.	64.8 ± 10.11
	Ranibizumab 0.5 mg + laser	118	64.0 ± 8.15	n. a.	70 (59.3)	48 (40.7)	n. a.	15 (12.7)	102 (86.4)	n. a. ^o^	14.62 ± 9.84	1.99 ± 3.14	n. a.	63.4 ± 9.99
	Laser + sham injection	111	63.5 ± 8.81	n. a.	58 (52.3)	53 (47.7)	n. a.	13 (11.7)	97 (87.4)	n. a. ^o^	12.93 ± 9.02	1.58 ± 1.96	n. a.	62.4 ± 11.11
VISTA [48]	Laser photo-coagulation + sham injection	154	61.7 ± 8.7	n. a.	n. a.	69 (44.8)	n. a.	n. a.	n. a.	7.6 ± 1.7	17.2 ± 9.5	n. a.	n. a.	59.7 ± 10.9
	Aflibercept 2 mg 2q4	154	62.0 ± 11.2	n. a.	n. a.	67 (43.5)	n. a.	n. a.	n. a.	7.9 ± 1.6	16.5 ± 9.9	n. a.	n. a.	58.9 ± 10.8
	Aflibercept 2 mg 2q8	151	63.1 ± 9.4	n. a.	n. a.	73 (48.3)	n. a.	n. a.	n. a.	7.9 ± 1.6	17.6 ± 11.5	n. a.	n. a.	59.4 ± 10.9
VIVID [48]	Laser photo-coagulation + sham injection	132	63.9 ± 8.6	n. a.	n. a.	54 (40.9)	n. a.	n. a.	n. a.	7.7 ± 1.3	14.5 ± 9.8	n. a.	n. a.	60.8 ± 10.6
	Aflibercept 2 mg 2q4	136	62.6 ± 8.6	n. a.	n. a.	53 (39.0)	n. a.	n. a.	n. a.	7.8 ± 1.5	14.3 ± 9.2	n. a.	n. a.	60.8 ± 10.7
	Aflibercept 2 mg 2q8	135	64.2 ± 7.8	n. a.	n. a.	47 (34.8)	n. a.	n. a.	n. a.	7.7 ± 1.4	14.1 ± 8.9	n. a.	n. a.	58.8 ± 11.2
DRCR.net Protocol T [49] ^p^	Aflibercept	224	60 ± 10	n. a.	n. a.	110 (49)	31.8 (27.4, 37.3) ^f^	22 (10)	196 (88)	7.6 (6.8, 9.1) ^f^	15 (8, 21) ^f^	n. a.	n. a.	69 (59, 74) ^f^
	Bevacizumab	218	62 ± 10	n. a.	n. a.	103 (47)	32.9 (28.7, 37.6) ^f^	12 (6)	205 (94)	7.7 (6.8, 8.8) ^f^	17 (11, 24) ^f^	n. a.	n. a.	69 (60, 72) ^f^
	Ranibizumab	218	60 ± 11	n. a.	n. a.	94 (43)	32.3 (28.2, 37.2) ^f^	16 (7)	196 (90)	7.8 (6.9, 9.2) ^f^	16 (11, 23) ^f^	n. a.	n. a.	68 (58, 73) ^f^

^a^ Method of measuring baseline VA not always explained in sources. Direct comparisons of VA results may not be reliable

^b^ Missing values in OCEAN: age: 9 patients; gender: 6; BMI: 87; diabetes type: 69; HbA1c: 491; time since diagnosis: 65; baseline VA: 8

^c^ Time since diagnosis until first injection in OCEAN

^d^ Results converted to years (from days)

^e^ Missing values in Protocol J (sham, ranibizumab, triamcinolone group): HbA1c: 3, 10, 6 patient (s)

^f^ Given as median (25th, 75th percentile)

^g^ Missing values in Protocol I (sham, ranibizumab + prompt laser, ranibizumab + deferred laser, triamcinolone group): HbA1c: 17, 3, 7, 8 patient(s)

^h^ No percentage available

^i^ Results converted to years (from months)

^k^ Missing values in RISE (sham, 0.3 mg, 0.5 mg group): BMI: 3, 3, 1 patient(s); time since diagnosis of diabetes: 4, 7, 7; HbA1c: 4, 5, 5; time since diagnosis of DME: 0, 1, 2

^m^ Time from clinically significant macular oedema to randomization

^n^ Missing values in RIDE (sham, 0.3 mg, 0.5 mg group): BMI: 2, 0, 1 patient(s); time since diagnosis of diabetes: 8, 6, 3; HbA1c: 5, 5, 4; time since diagnosis of DME: 0, 0, 1

^o^ HbA1c ≤10% inclusion criterion

^p^ Missing values in Protocol T (aflibercept, bevacizumab, ranibizumab group): diabetes type: 6, 1, 6 patients; HbA1c: 5, 0, 1 patient(s); BMI: 17, 22, 23 patients

Abbreviations: *2q4*: monthly until week 48; *2q8*: monthly until week 16 then every 8 weeks through week 48; *BMI*: body mass index; *DME*: diabetic macular oedema; *DRCR.net*: Diabetic Retinopathy Clinical Research Network; *ETDRS*: Early Treatment Diabetic Retinopathy Study; *HbA1c*: glycated haemoglobin (type A1c); *N*: total number of patients; n: number of patients; n. a.: data not available in source; *SD*: standard deviation; *VA*: visual acuity

The gender distribution in OCEAN (698 male DME patients, 57.6%, 95% CI [54.8; 60.4] %; 507 female patients, 41.9%, 95% CI [39.1; 44.7] %) was similar to that in the RCTs, with slightly more males than females included across all DME studies (Fig. 3).

The mean baseline VA (ETDRS letters ± SD) was 60.6 ± 15.5 letters (95% CI [59.7; 61.5] letters) for the OCEAN patients. VA was higher than this in RESTORE (mean between 62.4 and 64.8 letters) with a statistically significant difference to OCEAN for two of three treatment arms (non-overlapping 95% CIs). In contrast, baseline VA was significantly lower than in OCEAN for BOLT, RISE and RIDE (mean ranging from 54.6 to 57.5 letters; 95% CIs non-overlapping with OCEAN; Fig. 4). VA was reported as medians in the DRCR.net trials and was, therefore, not included in the statistical analysis.

The DME patients’ mean BMI in OCEAN was 29.3 ± 5.2 kg/m^2^ (95% CI [29.0; 29.6] kg/m^2^). This was statistically significantly lower than in RISE and RIDE, the only RCTs for which mean BMI data were available (means ranging from 31.3 to 32.9 kg/m^2^ for all treatment arms; 95% CIs non-overlapping with OCEAN).

The majority of DME patients in OCEAN (936 patients, 77.3%, 95% CI [74.8; 79.6] %) were reported to have type 2 diabetes (diabetes type unknown/missing for 69 OCEAN patients). This proportion was even higher (≥82%) in all RCTs for which data on the diabetes type were available. This difference compared to OCEAN reached statistical significance for DRCR.net Protocol I and Protocol T and for two of three treatment arms in RESTORE (95% CIs non-overlapping with OCEAN). No statistical difference was seen between OCEAN and the RCTs regarding the proportion of patients with type I diabetes, which was documented for 116 patients (9.6%, 95% CI [8.0; 11.4] %) in OCEAN.

The patients’ mean baseline level of HbA1c was 7.5% in OCEAN (95% CI [7.41; 7.59] %). This was similar or slightly higher across all trials (means ranging between 7.5% and 7.9%), for which these data were available. Notably, for two of three treatment arms in VISTA, the HbA1c values were statistically significantly higher than in OCEAN (non-overlapping 95% CIs).

The mean time (± SD) since the initial diagnosis of DME was under 1 year (0.7 ± 1.6 years; 95% CI [0.59; 0.77] years) for the OCEAN patients (range from 0 to 16 years) and higher in the RCTs for which this information was available (RESTORE, RIDE, RISE; means between 1.6 and 2.4 years). Due to the high SDs compared to the means for the time since diagnosis, the data do not allow a reliable evaluation. The mean time since the initial diagnosis of diabetes was available for all of the RCTs (range from 13 to 18 years), but not evaluated in OCEAN (Table 3).

### Comparison of OCEAN and RCTs for BRVO and CRVO

The comparison of the 204 BRVO patients from OCEAN with those from the two RCTs BRAVO and VIBRANT in this indication (for details see Table 4 and Additional file 7: Table S7) showed that the OCEAN patients were significantly older (mean ± SD: 71.2 ± 10.0 years) than those in the RCTs (mean ranging from 63.9 to 67.5 years; 95% CIs non-overlapping with OCEAN; Fig. 2).

**Table 4:** Patient demographics for OCEAN and selected randomized controlled trials in the indication retinal vein occlusion

Study	Treatment group	*N*	Age mean ± SD (years)	Age range (years)	Gender, *n* (%)		Time since first diagnosis of RVO mean ± SD (months)	Time since first diagnosis of RVO median [range] (months)	Baseline VA
					male	female			ETDRS letters analogue (mean ± SD)^a^
Indication BRVO									
OCEAN^b^ BRVO patients	Ranibizumab 0.5 mg	204	71.2 ± 10.0	39.2-92.7	85 (41.7)	119 (58.3)	6.30 ± 16.35 ^c,d^	1.63 [0–127] ^c,d^	55.9 ± 20.9
BRAVO [50]	Sham injections (6 months)	132	65.2 ± 12.7	26-89	74 (56.1)	58 (43.9)	3.7 ± 3.7	2 [0–16]	54.7 ± 12.2
	Ranibizumab 0.3 mg	134	66.6 ± 11.2	43-90	67 (50.0)	67 (50.0)	3.6 ± 4.1	2 [0–35]	56.0 ± 12.1
	Ranibizumab 0.5 mg	131	67.5 ± 11.8	41-91	71 (54.2)	60 (45.8)	3.3 ± 3.1	2 [0–13]	53.0 ± 12.5
VIBRANT [51]	Laser	90	63.9 ± 11.4	n. a.	n. a.	36 (40.0)	1.4 ± 1.3 ^d^	n. a.	57.7 ± 11.3
	Aflibercept 2 mg	91	67.0 ± 10.4	n. a.	n. a.	47 (51.6)	1.4 ± 1.4 ^d^	n. a.	58.6 ± 11.4
Indication CRVO									
OCEAN^b^ CRVO patients	Ranibizumab 0.5 mg	121	70.3 ± 11.5	26.7-91.9	57 (47.1)	64 (52.9)	3.78 ± 6.49 ^c,d^	1.44 [0–38] ^c,d^	43.7 ± 25.0
CRUISE [52]	Sham injections	130	65.4 ± 13.1	20-91	72 (55.4)	58 (44.6)	2.9 ± 2.9	2 [0–14]	49.2 ± 14.7
	Ranibizumab 0.3 mg	132	69.7 ± 11.6	38-90	71 (53.8)	61 (46.2)	3.6 ± 3.2	2 [0–12]	47.4 ± 14.8
	Ranibizumab 0.5 mg	130	67.6 ± 12.4	40-91	80 (61.5)	50 (38.5)	3.3 ± 3.7	2 [0–27]	48.1 ± 14.6
COPERNICUS [53]^e^	Aflibercept 2 mg 2q4, then PRN	114	65.5 ± 13.57	n. a.	69 (61)	45 (39)	2.73 ± 3.09	n. a.	50.7 ± 13.9
	Sham injections 2q4, then Aflibercept 2 mg PRN	73	67.5 ± 14.29	n. a.	38 (52)	35 (48)	1.88 ± 2.19	n. a.	48.9 ± 14.4
GALILEO [54]	VEGF Trap-Eye (Aflibercept) 2q4	103	59.9 ± 12.4	n. a.	58 (56.3)	45 (43.7)	2.6 ± 2.9^d^	n. a.	53.6 ± 15.8
	Sham injections	68	63.8 ± 13.3	n. a.	37 (54.4)	31 (45.6)	2.9 ± 2.6^d^	n. a.	50.9 ± 15.4

^a^Method of measuring baseline VA not always explained in sources. Direct comparisons of VA results may not be reliable

^b^Missing values in OCEAN: time since first diagnosis of RVO: 3 in BRVO, 3 in CRVO; baseline VA: 1 in BRVO, 3 in CRVO

^c^Time since diagnosis until first injection in OCEAN

^d^Results converted to months (originally in days)

^e^Missing values in COPERNICUS: Time since first diagnosis of RVO: 1 patient (in treatment group “Aflibercept 2 mg 2q4, then PRN”)

Abbreviations: *2q4*: every 4 weeks for 24 weeks; *BRVO*: branch retinal vein occlusion; *CRVO*: central retinal vein occlusion; *ETDRS*: Early Treatment Diabetic Retinopathy Study; *N*: total number of patients; *n*: number of patients; n. a.: data not available in source; *PRN*: pro re nata (as needed); *RVO*: retinal vein occlusion; *SD*: standard deviation; *VA*: visual acuity

The OCEAN study included a higher percentage of female patients (119 patients, 58.3%, 95% CI [51.2; 65.2] %) than male patients (85 patients, 41.7%, 95% CI [34.8; 48.8] %), while the situation was reversed in the RCTs, with a higher percentage of males in most treatment arms (Fig. 3). This difference reached statistical significance in one of two treatment arms of VIBRANT, with 60.0% male patients (95% CI [49.1; 70.2] %, non-overlapping with OCEAN).

The mean baseline VA was similar across the trials (ranging from 53 to 58.6 letters), with a VA of 55.9 ± 20.9 letters (95% CI [53.0; 58.8] letters) reported for the OCEAN patients (Fig. 4).

BMI data were only available for OCEAN (mean ± SD: 27.1 ± 4.3 kg/m^2^), therefore no comparison was possible.

The mean time since diagnosis of RVO was longest in OCEAN at around 6.3 months and shorter in the RCTs (mean between 1.4 and 3.7 months), while the median time since diagnosis was 1.6 months in OCEAN and around 2 months in BRAVO (no median available for VIBRANT).

The 121 CRVO patients in OCEAN were compared with those from the RCTs CRUISE, COPERNICUS and GALILEO. The OCEAN patients were the oldest, with a mean age of 70.3 ± 11.5 years (95% CI [68.3; 72.3] years), while the mean age ranged from 59.9 to 69.7 years in the RCTs (Fig. 2). This difference compared to OCEAN reached statistical significance for GALILEO and for one of the treatment arms in CRUISE and COPERNICUS, respectively (95% CIs non-overlapping with OCEAN).

Slightly more than half of the OCEAN CRVO patients were female (64 patients, 52.9%, 95% CI [43.6; 62.0] %), while all three RCTs included a higher percentage of male patients compared to females (range from 52% to 61.5% males; Fig. 3). However, this difference was not statistically significant.

The mean baseline VA of the OCEAN patients (ETDRS letters ± SD: 43.7 ± 25.0 letters, 95% CI [39.2; 48.2] letters) was slightly lower than in the RCTs (range from 47.4 to 53.6 letters; Fig. 4), but this difference was not significant overall.

BMI data were only available for the OCEAN patients (mean ± SD: 26.8 ± 4.4 kg/m^2^).

The mean time since diagnosis of RVO was around 3.8 months in OCEAN and ranged from 1.9 months to 3.6 months across the RCTs. Due to the high SDs compared to the means for the time since diagnosis, the data do not allow a reliable evaluation. The median time since diagnosis was 1.4 months for OCEAN and 2 months for CRUISE; no median was available for the COPERNICUS and GALILEO patients (Table 4).

## Discussion

### Assessment of the OCEAN study populations

The present analysis of the demographic data demonstrates the differences between the nAMD, DME and RVO populations of the OCEAN study. The mean patient age was significantly higher in the indication nAMD (77.9 years) than in the other indications, and significantly lower in DME compared to the other indications. This is in line with results from recent epidemiological studies: A global meta-analysis of patients with diabetic retinopathy (including DME but also milder forms) found a mean patient age of 58.1 years, reflecting the established fact that diabetic retinopathies including DME can affect relatively young patients [12]. In contrast, a retrospective chart review study conducted in Germany found a mean patient age of 77.5 years in the indication nAMD [19].

In OCEAN, more male than female patients with DME and more female than male patients with nAMD and RVO were enrolled. Again, this largely reflects epidemiological findings: The Gutenberg Health Study found that, in Germany, the incidence of diabetic retinopathy (DME or milder forms) was similar in both genders, but the incidence of diabetes mellitus itself was higher among males than females [20, 21]. A higher incidence of DME in males compared to females, as seen in OCEAN, may be founded in a gender bias in the incidence of underlying diabetes. In nAMD, two epidemiological studies found a higher incidence of disease in females compared to males [10, 19], as in OCEAN, while the Gutenberg Health Study found no correlation between nAMD incidence and gender in Germany [11]. In RVO, the Gutenberg Health Study found that males were affected more frequently than females [22] and this tendency was also seen in the RCTs analysed here. In contrast, the OCEAN RVO population included more females than males.

For all three OCEAN patient populations/indications, the mean BMI was in the overweight range, according to the World Health Organisation (WHO) BMI classification [23]. This is in line with the BMI published for the general population in Germany, particularly in the higher age groups where the BMI tends to be higher [24]. The mean BMI was significantly higher in the DME patients (29.3 kg/m^2^) than in all other indications. This supports findings from the German Gutenberg Health Study, where the median BMI of the diabetic population (with or without DME) was 30.7 kg/m^2^ [20]. As the nAMD patients in OCEAN were the oldest group but had a low mean BMI, these data are not in line with the fact that the BMI in the general population is known to increase with age. In this case, the co-morbidities, especially diabetes, likely influence the BMI more than the patients’ age.

The baseline VA was significantly better in the OCEAN DME population compared to the other indications, and it was significantly worse in the CRVO patients compared to all others. Considering the differences in VA results in each indication, it can be postulated that the OCEAN patients appear to have requested and started treatment comparably early in DME and BRVO and later in CRVO and nAMD, when VA had already deteriorated. The mean time since the diagnosis of the primary indication ranged between 0.3 and 0.7 years across the indications.

Overall, it should be taken into consideration that the OCEAN patient populations are not uniform cohorts. Considerable differences exist, for example, between DME patients with type 1 and type 2 diabetes and between BRVO and CRVO patients. While stratification of the existing data according to diabetes type was not feasible, the distinction between the two types of RVO was made in this analysis, as far as possible.

### Comparison of real-world OCEAN population with randomized controlled trials

In the literature-based analysis presented here, a number of differences were found between OCEAN and individual RCTs in the different indications. The results highlight how, for some parameters, single studies may differ considerably from the characteristics seen in other RCTs or the real-world cohort, while other parameters are similar among all RCTs and notably different in OCEAN. This may reflect differences in inclusion criteria. In the observational OCEAN study, inclusion and treatment were at the treating physician’s discretion, while the RCTs had narrower inclusion and exclusion criteria. These criteria included restrictions regarding baseline VA (e.g. >20/320), permitted pre-treatments, concomitant eye diseases and blood pressure, as well as indication-specific criteria like age restrictions and lesion characteristics in nAMD studies, restrictions regarding HbA1c levels, central subfield thickness and renal insufficiency in DME studies, and restrictions regarding central subfield thickness and specific concomitant diagnoses in RVO.

### Comparison of OCEAN and RCTs for nAMD

In nAMD, the most notable differences between OCEAN and the RCTs were seen in the patients’ age and baseline VA. The mean age of the OCEAN patients in nAMD was significantly higher than in some of the RCTs and lower than in others. In line with this, the age range was broader in OCEAN (36 to 101 years) than in the RCTs; this is unsurprising as the inclusion and exclusion criteria of all RCTs except for HARBOR did not permit the inclusion of patients under 50 years of age. The likelihood of misdiagnoses (idiopathic CNV, central serous chorioretinopathy) or atypical AMD might increase with younger age, while the diagnostic criteria are not as precisely defined as in typical nAMD cases. As the typical nAMD patient is of older age, none of the studies included a maximum age for eligibility, therefore allowing very elderly patients, with a potentially higher frequency of co-morbidities, to participate. The OCEAN patients’ mean VA at baseline was significantly better than in some of the RCTs and worse than in others. Regarding the patients’ medical history, cases of prior hypertension, prior myocardial infarction, stroke or apoplexy, or transient ischemic attack were reported less frequently in OCEAN than in most of the RCTs. This is most likely due to the non-interventional character associated with a less complete and strict reporting of historical data. Data on BMI were only available for OCEAN, where the mean BMI indicated an overweight nAMD population.

### Comparison of OCEAN and RCTs for DME

The OCEAN population in the indication DME differed from the respective RCTs regarding age. The OCEAN patients were significantly older than the patients in most of the RCTs, reflecting the less strict patient inclusion criteria. The OCEAN patients’ baseline VA was significantly better than in three of the RCTs. The DME patients’ mean BMI in OCEAN was in the overweight range, while it was significantly higher (obese) in RISE/RIDE. No information on the mean BMI was available for the other RCTs, which is an important drawback as obesity is a determinant of insulin resistance and a direct risk factor for developing diabetes and DME. However, in most of the RCTs, patients with high blood pressure or a history of cardiovascular events were excluded from the trials, therefore likely excluding highly obese patients. The percentage of patients with type 2 diabetes was slightly lower in OCEAN compared with the RCTs and also lower than in the general population in Germany, where an estimated 95% of all diabetes cases are type 2 [20, 25]. However, this may also be due to the proportion of OCEAN DME patients (approx. 6%) with unknown diabetes type. Previous studies showed that the type of diabetes did not impact the therapeutic response to DME treatment [26]. The HbA1c levels of the OCEAN patients were comparable to those of most of the RCTs, indicating that metabolic control was relatively good in these patients, although they were not as strictly selected as they may have been in the RCTs. However, the OCEAN cohort may also not be entirely representative and it is possible that patients with disease control issues were not recruited as often as patients with good compliance. The mean time since the initial diagnosis of DME was shorter for the OCEAN patients than for the patients in the RCTs for which this information was available. The mean time since the diagnosis of the underlying diabetes was not documented for the OCEAN patients; however, this parameter is an important factor when assessing a patient’s risk of progression. The fact that this parameter was not collected for the OCEAN patients reflects how some information is difficult to obtain in routine clinical practice, especially for long-term parameters that may not be readily available from a patient’s current file.

### Comparison of OCEAN and RCTs for BRVO and CRVO

In BRVO and CRVO, the OCEAN patients differed notably from those of the RCTs regarding age: The OCEAN patients were significantly older than those of most of the RCTs, again probably due to broader patient inclusion criteria for OCEAN. Further small differences were seen between the studies, although they mostly did not reach statistical significance: The percentage of females tended to be higher in OCEAN than in the RCTs. The mean baseline VA was similar for the BRVO patients in OCEAN and the RCTs, while the mean VA for CRVO patients was slightly worse in OCEAN than in the RCTs. The mean time since diagnosis of RVO tended to be longer in OCEAN than in the RCTs, while the median time since diagnosis was shorter in OCEAN than in the RCTs for which this parameter was available.

### Age as a risk factor for ocular diseases

Across the indications, the OCEAN patients tended to be older than those in the RCTs. It should be noted how the less strict inclusion and exclusion criteria of the NIS allow the inclusion of younger and older patients compared with some of the RCTs, where certain age criteria had to be fulfilled. As the general population ages and drugs are known to affect geriatric, co-morbid, co-medicated patients in different ways than patients of a middle age, more clinical and observational trials in older patients are warranted and will become important in future [27]. It can also be seen from analysis of the OCEAN data that older populations, like the nAMD patient group, include more females than males, which may reflect the generally higher life expectancy of women in Europe [28]. In spite of the large differences between the ophthalmological indications treated with anti-VEGF drugs, most of these indications are age-related diseases. Particular requirements and potential risks in the older population have to be taken into account when administering intravitreal drugs over longer periods of time. Certain factors cause an under-representation of particularly old patients in trials and co-morbidities and underutilization of treatments, e.g. in care homes, might influence the risk-benefit assessment of anti-VEGF treatments. However, an effective treatment preventing blindness should be offered to each patient with a relevant expected benefit, irrespective of the patient’s age.

### Complementarity of NISs and RCTs

Across the trials and indications, the results from OCEAN tended to show higher or similar SDs than the RCT data, pointing to the broader variation in patient characteristics allowed in a NIS. While the divergent characteristics limit the scientific conclusions regarding drug effectiveness, they allow assessments of patient responses outside of the typical patient profile. The average patient for a particular treatment is usually enrolled and assessed in RCTs, but a less typical patient type, who may not be eligible for an RCT, can be observed in a NIS. This may even lead to an expansion of the treatment scope for a particular therapy, if interesting effectiveness or safety findings from a NIS are followed up and confirmed in RCTs. A good example of such a situation is the number of young patients included in OCEAN for treatment of nAMD. In the RCTs for nAMD, the minimum patient age was usually 50 years. It is not clear whether this reflects misdiagnoses (e.g. central serous chorioretinopathy) or natural variability of the disease. This and other strict inclusion and exclusion criteria achieve a confinement of the patient population to include mainly typical cases, which increases the likelihood of a correct diagnosis beforehand and of comparable study outcomes. Such a controlled design helps to define the gold standard for treatment, but atypical cases and confounding pathologies are usually and purposefully excluded. As opposed to this, the less strict selection criteria of NISs can decrease a potential selection bias and the results of such NISs allow conclusions regarding the general population. In spite of the frequent loss of follow-up, NISs such as OCEAN provide real-world evidence and are powered to observe rare events, as they often include larger patient populations than RCTs. The longer time frames of data collection and interpretation of NISs in comparison with RCTs particularly lend themselves to improving the understanding of complex long-term diseases. In addition to allowing an assessment of the effectiveness and safety of a drug in a setting that is reflective of clinical practice, NISs can also complement the evidence base from RCTs by gauging the benefits across a more diverse range of outcome measures. These include comparative effectiveness data between multiple therapies when equivalent data from RCTs are not available, as well as information on long-term benefit-risk profiles, patient experience, patient-reported outcomes (PROs) and economic outcomes [1, 2]. Despite the differences between RCTs and NISs, both are important for improving our understanding of disease outcomes and treatment effects, and the two methodologies should be viewed as complementary rather than competing.

The comparative analysis presented here confirms how an ophthalmological NIS like OCEAN can provide data that are generally in line with the RCTs in the same field, but that include broader populations. A limitation of this analysis was that the literature review focussed on larger studies, in order to keep the number of studies in a manageable range for the comparisons to OCEAN. Furthermore, the exact method of the respective VA assessments was not always explained in detail in the source publications, therefore the comparison of VA results across the studies has to be treated with caution. In addition, the OCEAN study was limited to Germany, while the RCTs were conducted in other countries world-wide. Comparing the OCEAN data to epidemiological data from Germany shows that the OCEAN populations appear to be relatively close to what is seen for the general population. In addition, the RCTs included in the present study were largely conducted in Europe or the USA and included a majority of white/Caucasian patients. Thus, the OCEAN results should be comparable and not subject to ethnicity-driven bias. Although a NIS like OCEAN is care-driven and physicians were asked to include all patients who were eligible and consented, a selection bias cannot be excluded. As the main focus of this paper was on the baseline demographic parameters, it is unlikely that the data are affected by the Hawthorne effect, i.e. by a change in patient and/or physician behaviour because they know that they are being observed in a trial [29].

## Conclusions

The similarities as well as the differences that were noted between baseline demographic characteristics of the OCEAN patients and patients from the selected RCTs underline the complementarity of NISs and RCTs. The OCEAN patient population covers a broader spectrum of patient profiles than RCTs. While RCT results allow analyses based on precisely defined settings and complete datasets, NISs provide valuable information that can help guide patient management in the real-world setting, based on larger and more heterogeneous patient collectives than RCTs. Patient sub-groups and non-typical cases can be included in NISs, for a representative sample of the real-world population.

The differences that were seen in the present analysis related to age parameters, across the trials but also across the indications, indicate that ophthalmology often deals with patient subgroups at the extremes of the age range, i.e. very old and atypically young patients. Research into treatment requirements and issues for these patient groups has to be explored by NISs, for which, therefore, there is a continued need. However, missing or incomplete data sets, the non-blinded setting and potential selection biases have to be taken into account for a NIS like OCEAN. Future NISs should be designed with these issues in mind. As far as possible in a non-interventional setting, data collection strategies should be optimized further; case report forms must be as simple as possible, while allowing precise documentation of complex situations, especially if physicians’ data entries are not queried. Ideally, data should be documented directly in an electronic database. A strength of NISs is the fact that larger patient populations can be included than in the usual RCTs. Here, representativeness of the results can be maximized by including a large number of patients, as was done in OCEAN. However, OCEAN is limited to Germany, whereas most of the RCTs discussed are multi-national trials.

## Additional files

Additional file 1: Table S1. Table of confidence intervals for baseline demographic characteristics of OCEAN patients, by indication. (DOCX 14 kb)
Additional file 2: Table S2. Table of selected randomized controlled trials for comparison to OCEAN, by indication. (DOCX 15 kb)
Additional file 3: Table S3. Table of confidence intervals for baseline demographic characteristics (age, gender, time since diagnosis, baseline visual acuity) in the indication neovascular age-related macular degeneration: results for the OCEAN study and for selected randomized controlled trials. (DOCX 25 kb)
Additional file 4: Table S4. Table of confidence intervals for medical history parameters in the indication neovascular age-related macular degeneration: results for the OCEAN study and for those selected randomized controlled trials for which comparable data were available. (DOCX 15 kb)
Additional file 5: Table S5. Table of confidence intervals for baseline demographic characteristics (age, gender, time since diagnosis, baseline visual acuity) in the indication diabetic macular oedema: results for the OCEAN study and for selected randomized controlled trials. (DOCX 20 kb)
Additional file 6: Table S6. Table of confidence intervals for additional baseline demographic parameters (BMI, diabetes type, HbA1c) in the indication diabetic macular oedema: results for the OCEAN study and for selected randomized controlled trials. (DOCX 18 kb)
Additional file 7: Table S7. Table of confidence intervals for baseline demographic characteristics (age, gender, time since diagnosis, baseline visual acuity) in the indications branch and central retinal vein occlusion: results for the OCEAN study and for selected randomized controlled trials. (DOCX 20 kb)

## Abbreviations

BMI: Body mass index
BRVO: Branch retinal vein occlusion
CI: Confidence interval
CRVO: Central retinal vein occlusion
DME: Diabetic macular oedema
DRCR.net: Diabetic Retinopathy Clinical Research Network
ETDRS: Early Treatment Diabetic Retinopathy Study
HbA1c: Glycated haemoglobin (type A1c)
nAMD: Neovascular age-related macular degeneration
NIS: Non-interventional study
OCT: Optical coherence tomography
PRN: Pro re nata (as needed)
RCT: Randomized controlled trial
RVO: Retinal vein occlusion
SD: Standard deviation
VA: Visual acuity
VEGF: Vascular endothelial growth factor
WHO: World Health Organisation
