# Supplementary material for: Demographics of patients receiving Intravitreal anti-VEGF treatment in real-world practice: healthcare research data versus randomized controlled trials
Source: BMC Ophthalmol. 2017 Jan 19;17:7. doi: 10.1186/s12886-017-0401-y (PMC5244516; doi:10.1186/s12886-017-0401-y)
Supplement: Additional file 1: Table S1. — Table of confidence intervals for baseline demographic characteristics of OCEAN patients, by indication. (DOCX 14 kb) [file 12886_2017_401_MOESM1_ESM.docx]

### **Additional File 1**

### **Table S1** Table of confidence intervals for baseline demographic characteristics of OCEAN patients, by indication.

| **Primary indication** | **N** | **Age** | | **Gender** | | | | **BMI** | | **Time since diagnosis of primary indication** ^a^ | | **Baseline VA** | |
| --- | --- | --- | --- | --- | --- | --- | --- | --- | --- | --- | --- | --- | --- |
|  |  | Mean ± SD (years) | 95% CI (years) | Males,  n (%) | Males,  95% CI (%) | Females, n (%) | Females,  95% CI (%) | Mean ± SD (kg/m^2^) | 95% CI (kg/m^2^) | Mean ±SD (years) | 95% CI (years) | ETDRS letters (mean±SD) | 95% CI (letters) |
| nAMD ^b^ | 3614 | 77.9 ± 8.2 | [77.6; 78.2] | 1393 (38.5) | [36.9; 40.1] | 2210 (61.2) | [59.5; 62.7] | 26.6 ± 4.0 | [26.47; 26.73] | 0.53 ± 1.28 | [0.49; 0.57] ^c^ | 52.0 ± 21.3 | [51.3; 52.7] |
| DME ^d^ | 1211 | 67.6 ± 10.9 | [67.0; 68.2] | 698 (57.6) | [54.8; 60.4] | 507  (41.9) | [39.1; 44.7] | 29.3 ± 5.2 | [29.01; 29.59] | 0.68 ± 1.63 | [0.59; 0.77] ^c^ | 60.6 ± 15.5 | [59.7; 61.5] |
| BRVO ^e^ | 204 | 71.2 ± 10.0 | [69.8; 72.6] | 85 (41.7) | [34.8; 48.8] | 119  (58.3) | [51.2; 65.2] | 27.1 ± 4.3 | [26.51; 27.69] | 0.53 ± 1.37 | [0.31; 0.69] ^c^ | 55.9 ± 20.9 | [53.0; 58.8] |
| CRVO ^f^ | 121 | 70.3 ± 11.5 | [68.3; 72.3] | 57 (47.1) | [38.0; 56.4] | 64  (52.9) | [43.6; 62.0] | 26.8 ± 4.4 | [26.02; 27.58] | 0.32 ± 0.54 | [0.22; 0.42] ^c^ | 43.7 ± 25.0 | [39.2; 48.2] |
| ^a^ Time since diagnosis of primary indication until first injection in OCEAN study.  ^b^ nAMD: Missing values: age: 14 patients; gender: 11; BMI: 230; time since diagnosis: 199; baseline VA: 29.  ^c^ Calculation of CIs based on an approximation assuming normal distribution; limited reliability of results due to high SD compared to mean.  ^d^ DME: Missing values: age: 9 patients; gender: 6; BMI: 87; time since diagnosis: 65; baseline VA: 8.  ^e^ BRVO: Missing values: age and gender: 0 patients; BMI: 8; time since diagnosis: 3; baseline VA: 1.  ^f^ CRVO: Missing values: age and gender: 0 patients; BMI: 2; time since diagnosis: 3; baseline VA: 3.  Abbreviations: BRVO: branch retinal vein occlusion; CI: confidence interval; CRVO: central retinal vein occlusion; DME: diabetic macular oedema; ETDRS: Early Treatment Diabetic Retinopathy Study; N: total number of patients; n: number of patients; nAMD: neovascular age-related macular degeneration; SD: standard deviation; VA: visual acuity. | | | | | | | | | | | | | |
