# Supplementary material for: Demographics of patients receiving Intravitreal anti-VEGF treatment in real-world practice: healthcare research data versus randomized controlled trials
Source: BMC Ophthalmol. 2017 Jan 19;17:7. doi: 10.1186/s12886-017-0401-y (PMC5244516; doi:10.1186/s12886-017-0401-y)
Supplement: Additional file 2: Table S2. — Table of selected randomized controlled trials for comparison to OCEAN, by indication. (DOCX 15 kb) [file 12886_2017_401_MOESM2_ESM.docx]

### **Additional File 2**

### **Table S2**Table of selected randomized controlled trials for comparison to OCEAN, by indication.

| **Study** | **Number of patients** | **Study start and end dates*** | **Trial ID** |
| --- | --- | --- | --- |
| **Indication nAMD** |  |  |  |
| **MARINA** [30]: A Study to Evaluate rhuFab V2 [Ranibizumab] in Subjects With Minimally Classic or Occult Subfoveal Neovascular Macular Degeneration | 716 | Mar 2003 to Dec 2005 | Clinicaltrials.gov ID: NCT00056836 |
| **ANCHOR** [31]: A Study to Compare rhuFab V2 [Ranibizumab] With Verteporfin Photodynamic in Treating Subfoveal Neovascular Macular Degeneration | 423 | May 2003 to Sep 2004 (end of recruitment) | Clinicaltrials.gov ID: NCT00061594 |
| **PIER** [32]: A Study of rhuFab V2 (Ranibizumab) in Subjects With Subfoveal Choroidal Neovascularization Secondary to Age-Related Macular Degeneration | 184 | Aug 2004 to Mar 2007 | Clinicaltrials.gov ID: NCT00090623 |
| **SAILOR** [33]: A Study to Evaluate Ranibizumab in Subjects With Choroidal Neovascularization (CNV) Secondary to Age-Related Macular Degeneration | 2378 | Nov 2005 to Sep 2007 | Clinicaltrials.gov ID: NCT00251459 |
| **EXCITE** [34]: Efficacy and Safety of Ranibizumab in Patients With Subfoveal Choroidal Neovascularization (CNV) Secondary to Age-related Macular Degeneration (AMD) | 353 | Dec 2005 to Jan 2008 | Clinicaltrials.gov ID: NCT00275821 |
| **ABC** [35]: A randomised, double-masked phase III study of the efficacy and safety of Avastin^®^ (bevacizumab) intravitreal injections compared to best available therapy in subjects with choroidal neovascularisation secondary to age-related macular degeneration | 131 | Aug 2006 to  Dec 2008 | ISRCTN ID: 83325075 |
| **IVAN** [36]: A randomised controlled trial of alternative treatments to inhibit VEGF in age-related choroidal neovascularisation | 610 | Jul 2007 to  Nov 2012 | ISRCTN ID: 92166560 |
| **VIEW** [37]: Vascular Endothelial Growth Factor VEGF Trap-Eye: Investigation of Efficacy and Safety in Wet Age-Related Macular Degeneration (AMD) | VIEW1 + VIEW2: 2412 | VIEW 1: Aug 2007 to Sep 2010; VIEW2: Apr 2008 to Sep 2010 | Clinicaltrials.gov ID: NCT00509795 (VIEW1) and NCT00637377 (VIEW2) |
| **CATT** [38]: Comparison of Age-related Macular Degeneration Treatments Trials: Lucentis-Avastin Trial | 1107 | Feb 2008 to Dec 2010 | Clinicaltrials.gov ID: NCT00593450 |
| **MANTA** [39]: Avastin Versus Lucentis in Age Related Macular Degeneration | 317 | Jul 2008 to Dec 2011 | Clinicaltrials.gov ID: NCT00710229 |
| **LUCAS** [40]: Lucentis Compared to Avastin Study | 431 | Mar 2009 to Aug 2014 | Clinicaltrials.gov ID: NCT01127360 |
| **GEFAL** [41]: French Evaluation Group Avastin Versus Lucentis | 374 | Jun 2009 to Dec 2012 | Clinicaltrials.gov ID: NCT01170767 |
| **HARBOR** [42]: A Study of Ranibizumab Administered Monthly or on an As-needed Basis in Patients With Subfoveal Neovascular Age-related Macular Degeneration | 1097 | Jul 2009 to Aug 2011 | Clinicaltrials.gov ID: NCT00891735 |
| **Indication DME** |  |  |  |
| **DRCR.net** trial **Protocol J** [43]: Laser-Ranibizumab-Triamcinolone for Proliferative Diabetic Retinopathy | 319 patients; 345 eyes | Mar 2007 to Oct 2009 | Clinicaltrials.gov ID: NCT00445003 |
| **DRCR.net** trial **Protocol I** [44]: Laser-Ranibizumab-Triamcinolone for Diabetic Macular Edema | 691 patients; 854 eyes | Mar 2007 to Dec 2009 | Clinicaltrials.gov ID: NCT00444600 |
| **BOLT** [45]: Diabetic macular oedema: a prospective randomised trial of management with intravitreal bevacizumab versus conventional laser therapy | 80 | May 2007 to Aug 2009 (last patient last visit) | EudraCT ID: 2007-000847-89 |
| **RISE** [46]: A Study of Ranibizumab Injection in Subjects With Clinically Significant Macular Edema (ME) With Center Involvement Secondary to Diabetes Mellitus | 377 | Jun 2007 to Nov 2010 | Clinicaltrials.gov ID: NCT00473330 |
| **RIDE** [46]: A Study of Ranibizumab Injection in Subjects With Clinically Significant Macular Edema (ME) With Center Involvement Secondary to Diabetes Mellitus | 382 | Jun 2007 to Jan 2011 | Clinicaltrials.gov ID: NCT00473382 |
| **RESTORE** [47]: A 12 Month Core Study to Assess the Efficacy and Safety of Ranibizumab (Intravitreal Injections) in Patients With Visual Impairment Due to Diabetic Macular Edema and a 24 Month Open-label Extension Study | 345 | May 2008 to Jan 2010 | Clinicaltrials.gov ID: NCT00687804 |
| **VISTA** [48]: Study of Intravitreal Aflibercept Injection in Patients With Diabetic Macular Edema | 459 | May 2011 to Jan 2013 | Clinicaltrials.gov ID: NCT01363440 |
| **VIVID** [48]: Intravitreal Aflibercept Injection in Vision Impairment Due to DME | 403 | May 2011 to  Jun 2013 | Clinicaltrials.gov ID: NCT01331681 |
| **DRCR.net Protocol T** [49]: Comparative Effectiveness Study of Intravitreal Aflibercept, Bevacizumab, and Ranibizumab for DME | 660 | Aug 2012 to Oct 2014 | Clinicaltrials.gov ID: NCT01627249 |
| **Indication BRVO** |  |  |  |
| **BRAVO** [50]: A Study of the Efficacy and Safety of Ranibizumab Injection in Patients With Macular Edema Secondary to Branch Retinal Vein Occlusion | 397 | Jul 2007 to  May 2009 | Clinicaltrials.gov ID: NCT00486018 |
| **VIBRANT** [51]: Study to Assess the Clinical Efficacy and Safety of Intravitreal Aflibercept Injection in Patients With Branch Retinal Vein Occlusion | 181 | Apr 2012 to Aug 2013 | Clinicaltrials.gov ID: NCT01521559 |
| **Indication CRVO** |  |  |  |
| **CRUISE** [52]: A Study of the Efficacy and Safety of Ranibizumab Injection in Patients With Macular Edema Secondary to Central Retinal Vein Occlusion | 392 | Jul 2007 to  Jun 2009 | Clinicaltrials.gov ID: NCT00485836 |
| **COPERNICUS** [53]: Vascular Endothelial Growth Factor (VEGF) Trap-Eye: Investigation of Efficacy and Safety in Central Retinal Vein Occlusion | 187 | Jul 2009 to Oct 2010 | Clinicaltrials.gov ID: NCT00943072 |
| **GALILEO** [54]: Vascular Endothelial Growth Factor (VEGF) Trap-Eye: Investigation of Efficacy and Safety in Central Retinal Vein Occlusion | 171 | Oct 2009 to Feb 2011 | Clinicaltrials.gov ID: NCT01012973 |
| * Study end date was the “final data collection date for primary outcome measure” (as provided on clinicaltrials.gov) or the “overall trial end date” (as provided on ISRCTN website), unless stated otherwise  Sources: Studies with NCT ID numbers: information from www.clinicaltrials.gov (accessed on 07 Sep 2015). Studies with EudraCT ID: information from www.clinicaltrialsregister.eu (accessed on 07 Sep 2015). Studies with ISRCT ID: Information from www.controlled-trials.com (accessed on 07 Sep 2015)  Abbreviations: BRVO: branch retinal vein occlusion; CRVO: central retinal vein occlusion; DME: diabetic macular oedema; DRCR.net: Diabetic Retinopathy Clinical Research Network; ID: identification number; nAMD: neovascular age-related macular degeneration; RVO: retinal vein occlusion. | | | |
