# Supplementary material for: Demographics of patients receiving Intravitreal anti-VEGF treatment in real-world practice: healthcare research data versus randomized controlled trials
Source: BMC Ophthalmol. 2017 Jan 19;17:7. doi: 10.1186/s12886-017-0401-y (PMC5244516; doi:10.1186/s12886-017-0401-y)
Supplement: Additional file 3: Table S3. — Table of confidence intervals for baseline demographic characteristics (age, gender, time since diagnosis, baseline visual acuity) in the indication neovascular age-related macular degeneration: results for the OCEAN study and for selected randomized controlled trials. (DOCX 25 kb) [file 12886_2017_401_MOESM3_ESM.docx]

### **Additional file 3**

### **Table S3**Table of confidence intervals for baseline demographic characteristics (age, gender, time since diagnosis, baseline visual acuity) in the indication neovascular age-related macular degeneration: results for the OCEAN study and for selected randomized controlled trials.

| **Study** | **Treatment group** | **N** | **Age** | | **Gender** | | | | **Time since diagnosis of nAMD** | | **Baseline VA** | |
| --- | --- | --- | --- | --- | --- | --- | --- | --- | --- | --- | --- | --- |
|  |  |  | Mean ± SD (years) | 95% CI (years) | Males,  n (%) | Males,  95% CI (%) | Females n (%) | Females, 95% CI (%) | Mean ±SD (years) | 95% CI (years) | ETDRS letters analogue (mean ± SD) ^a^ | 95% CI (letters) |
| **OCEAN** ^b^ | Ranibizumab 0.5 mg | 3614 | 77.9 ± 8.2 | [77.6; 78.2] | 1393 (38.5) | [36.9; 40.1] | 2210 (61.2) | [59.5; 62.7] | 0.53 ± 1.28 ^c,d^ | [0.49; 0.57] ^e^ | 52.0 ± 21.3 | [51.3; 52.7] |
| MARINA [30] | Sham injections q4 | 238 | 77 ± 7 | [76.1; 77.9] | 79 (33.2) | [27.2; 39.6] | 159 (66.8) | [60.4; 72.8] | n. a. | n. a. | 53.6 ± 14.1 | [51.8; 55.4] |
|  | Ranibizumab 0.3 mg | 238 | 77 ± 8 | [76.0; 78.0] | 85 (35.7) | [29.6; 42.2] | 153 (64.3) | [57.8; 70.4] | n. a. | n. a. | 53.1 ± 12.9 | [51.5; 54.7] |
|  | Ranibizumab 0.5 mg | 240 | 77 ± 8 | [76.0; 78.0] | 88 (36.7) | [30.6; 43.1] | 152 (63.3) | [59.9; 69.4] | n. a. | n. a. | 53.7 ± 12.8 | [52.1; 55.3] |
| ANCHOR [31] ^f^ | Verteporfin + sham injections + laser | 143 | 77.7 ± 7.8 | [76.4; 79.0] | 64 (44.8) | [36.4; 53.3] | 79 (55.2) | [46.7; 63.6] | n. a. | n. a. | 45.5 ± 13.1 | [43.4; 47.6] |
|  | Ranibizumab 0.3 mg + sham verteporfin + laser | 140 | 77.4 ± 7.5 | [76.2; 78.6] | 73 (52.1) | [43.5; 60.7] | 67 (47.9) | [39.4; 56.5] | n. a. | n. a. | 47.0 ± 13.1 | [44.8; 49.2] |
|  | Ranibizumab 0.5 mg + sham verteporfin + laser | 140 | 76.0 ± 8.6 | [74.6; 77.4] | 75 (53.6) | [45.0; 62.0] | 65 (46.4) | [38.0; 55.1] | n. a. | n. a. | 47.1 ± 13.2 | [44.9; 49.3] |
| PIER [32] ^g^ | Sham | 63 | 77.8 ± 7.1 | [76.0; 79.6] | 20 (31.7) | [20.6; 44.7] | 43 (68.3) | [55.3; 79.4] | 0.3 ± 0.5 | [0.18; 0.42] ^e^ | 55.1 ± 13.9 | [51.7; 58.5] |
|  | Ranibizumab 0.3 mg | 60 | 78.7 ± 6.3 | [77.1; 80.3] | 26 (43.3) | [30.6; 56.8] | 34 (56.7) | [43.2; 69.4] | 0.7 ± 1.6 | [0.30; 1.10] ^e^ | 55.8 ± 12.2 | [52.7; 58.9] |
|  | Ranibizumab 0.5 mg | 61 | 78.8 ± 7.9 | [76.8; 80.8] | 28 (45.9) | [33.1; 59.2] | 33 (54.1) | [40.9; 66.9] | 0.7 ± 1.2 | [0.40; 1.00] ^e^ | 53.7 ± 15.5 | [49.8; 57.6] |
| SAILOR [33] ^h^ | Cohort 1 arm 1: Ranibizumab 0.3 mg | 1169 | 78.7 ± 7.6 | [78.3; 79.1] | 469 (40.1) ^i^ | [37.3; 43.0] | 700 (59.9) ^k^ | [57.0; 62.7] | 0.3 ± 1.4 ^m^  1.4 ± 2.0 ^n^ | [0.17; 0.43] ^m^  [1.25; 1.55] ^n,e^ | 55.0 ± 12.5 ^m^ 53.8 ± 13.8 ^n^ | [53.9; 56.1] ^m^  [52.8; 54.8] ^n^ |
|  | Cohort 1 arm 2: Ranibizumab 0.5 mg | 1209 | 78.7 ± 8.6 | [78.2; 79.2] | 507 (41.9) ^i^ | [39.1; 44.8] | 702 (58.1) ^k^ | [55.2; 60.9] | 0.3 ± 0.7 ^m^  1.3 ± 1.7 ^n^ | [0.24; 0.36] ^m^  [1.18; 1.42] ^n,e^ | 48.9 ± 13.8 ^m^ 50.0 ± 14.3 ^n^ | [47.7; 50.1] ^m^  [49.0; 51.0] ^n^ |
| EXCITE [34] | Ranibizumab 0.3 mg q16 | 120 | 75.1 ± 7.45 | [73.8; 76.4] | 50 (41.7) | [32.7; 51.0] | 70 (58.3) | [49.0; 67.3] | 0.57 ± 1.42 | [0.32; 0.82] ^e^ | 55.8 ± 11.81 | [53.7; 57.9] |
|  | Ranibizumab 0.5 mg q16 | 118 | 75.8 ± 6.96 | [74.5; 77.1] | 45 (38.1) | [29.4; 47.5] | 73 (61.9) | [52.5; 70.7] | 0.52 ± 1.14 | [0.31; 0.73] ^e^ | 57.7 ± 13.06 | [55.3; 60.1] |
|  | Ranibizumab 0.3 mg q4 | 115 | 75 ± 8.26 | [73.5; 76.5] | 49 (42.6) | [33.4; 52.2] | 66 (57.4) | [47.8; 66.6] | 0.56 ± 2.18 | [0.16; 0.96] ^e^ | 56.5 ± 12.19 | [54.3; 58.7] |
| ABC [35] | Bevacizumab 1.25 mg, q6 | 65 | n. a. | n. a. | 26 (40) | [28.0; 52.9] | 39 (60) | [47.1; 72.0] | n. a. | n. a. | n. a. | n. a. |
|  | Standard therapy ^o^ | 66 | n. a. | n. a. | 25 (38) | [26.2; 50.7] | 41 (62) | [49.3; 73.8] | n. a. | n. a. | n. a. | n. a. |
| IVAN [36] | Ranibizumab 0.5 mg | 314 | 77.8 ± 7.6 | [77.0; 78.6] | 129 (41) | [35.6; 46.8] | 185 (59) ^p^ | [53.3; 64.4] | n. a. | n. a. | 61.8 ± 15.0 | [60.1; 63.5] |
|  | Bevacizumab 1.25 mg | 296 | 77.7 ± 7.3 | [76.9; 78.5] | 115 (39) | [33.3; 44.7] | 181 (61) ^p^ | [55.3; 66.7] | n. a. | n. a. | 61.1 ± 15.5 | [59.3; 62.9] |
| VIEW [37] (VIEW1 + VIEW2 pooled) | Ranibizumab 0.5 mg q4 | 595 | 75.6 ± 8.7 | [74.9; 76.3] | 254 (42.7) ^i^ | [38.7; 46.8] | 341 (57.3) | [53.2; 61.3] | n. a. | n. a. | 53.9 ± 13.4 | [52.8; 55.0] |
|  | Aflibercept 2 mg q4 | 613 | 75.9 ± 8.4 | [75.2; 76.6] | 243 (39.6) ^i^ | [35.8; 43.6] | 370 (60.4) | [56.4; 64.3] | n. a. | n. a. | 54.0 ± 13.6 | [52.9; 55.1] |
|  | Aflibercept 0.5 mg q4 | 597 | 76.5 ± 8.5 | [75.8; 77.2] | 283 (47.4) ^i^ | [43.3; 51.5] | 314 (52.6) | [48.5; 56.7] | n. a. | n. a. | 53.6 ± 13.8 | [52.5; 54.7] |
|  | Aflibercept 2 mg q8 | 607 | 75.8 ± 8.8 | [75.1; 76.5] | 254 (41.8) ^i^ | [37.9; 45.9] | 353 (58.2) | [54.1; 62.1] | n. a. | n. a. | 53.6 ± 13.5 | [52.5; 54.7] |
| CATT [38] | Ranibizumab 0.5 mg q4 | 146 | 79.5 ± 7.4 | [78.3; 80.7] | 56 (38.4) | [30.4; 46.8] | 90 (61.6) | [53.2; 69.6] | n. a. | n. a. | 59.9 ± 14.2 | [57.6; 62.2] |
|  | Bevacizumab 1.25 mg q4 | 135 | 79.7 ± 7.5 | [78.4; 81.0] | 53 (39.3) | [31.0; 48.0] | 82 (60.7) | [52.0; 69.0] | n. a. | n. a. | 60.2 ± 13.6 | [57.9; 62.5] |
|  | Ranibizumab 0.5 mg PRN | 287 | 78.3 ± 7.8 | [77.4; 79.2] | 108 (37.6) | [32.0; 43.5] | 179 (62.4) | [56.5; 68.0] | n. a. | n. a. | 61.6 ± 13.1 | [60.1; 63.1] |
|  | Bevacizumab 1.25 mg PRN | 270 | 78.9 ± 7.4 | [78.0; 79.8] | 104 (38.5) | [32.7; 44.6] | 166 (61.5) | [55.4; 67.3] | n. a. | n. a. | 60.6 ± 13.0 | [59.0; 62.2] |
|  | Ranibizumab 0.5 mg year 1 q4, year 2 PRN | 138 | 78.8 ± 7.5 | [77.5; 80.1] | 56 (40.6) | [32.3; 49.3] | 82 (59.4) | [50.7; 67.7] | n. a. | n. a. | 60.9 ± 14.3 | [58.5; 63.3] |
|  | Bevacizumab 1.25 mg year 1 q4, year 2 PRN | 131 | 80.4 ± 7.1 | [79.2; 81.6] | 45 (34.4) | [26.3; 43.2] | 86 (65.6) | [56.9; 73.7] | n. a. | n. a. | 60.4 ± 12.4 | [58.3; 62.5] |
| MANTA [39] | Bevacizumab 1.25 mg | 154 | 76.7 ± 7.8 | [75.5; 77.9] | 56 (36.4) ^q^ | [28.8; 44.5] | 98 (63.6) ^q^ | [55.5; 71.2] | n. a. | n. a. | 57.0 ± 13.0 | [54.9; 59.1] |
|  | Ranibizumab 0.5 mg | 163 | 77.6 ± 8.1 | [76.4; 78.8] | 59 (36.2) ^q^ | [28.8; 44.1] | 104 (63.8) ^q^ | [55.9; 71.2] | n. a. | n. a. | 56.4 ± 13.5 | [54.3; 58.5] |
| LUCAS [40] | Bevacizumab 1.25 mg | 213 | 78.7 ± 7.6 | [77.7; 79.7] | 62 (29.1) | [23.1; 35.7] | 151 (70.9) | [64.3; 76.9] | n. a. | n. a. | 60 ± 14 | [58.1; 61.9] |
|  | Ranibizumab 0.5 mg | 218 | 78.0 ± 8.2 | [76.9; 79.1] | 78 (35.8) | [29.4; 42.5] | 140 (64.2) | [57.5; 70.6] | n. a. | n. a. | 62 ± 13 | [60.3; 63.7] |
| GEFAL [41] | Bevacizumab 1.25 mg | 191 | 79.62 ± 6.90 | [78.6; 80.6] | 72 (37.7) | [30.8; 45.0] | 119 (62.3) | [55.0; 69.2] | n. a. | n. a. | 54.62 ± 14.07 | [52.6; 56.6] |
|  | Ranibizumab 0.5 mg | 183 | 78.68 ± 7.27 | [77.6; 79.7] | 54 (29.5) | [23.0; 36.7] | 129 (70.5) | [63.3; 77.0] | n. a. | n. a. | 55.78 ± 13.99 | [53.8; 57.8] |
| HARBOR [42] | Ranibizumab 0.5 mg q4 | 275 | 78.8 ± 8.4 | [77.8; 79.8] | 113 (41.1) | [35.2; 47.2] | 162 (58.9) | [52.8; 64.8] | n. a. | n. a. | 54.2 ± 13.3 | [52.6; 55.8] |
|  | Ranibizumab 0.5 mg PRN | 275 | 78.5 ± 8.3 | [77.5; 79.5] | 112 (40.7) | [34.9; 46.8] | 163 (59.3) | [53.2; 65.1] | n. a. | n. a. | 54.5 ± 11.7 | [53.1; 55.9] |
|  | Ranibizumab 2.0 mg q4 | 274 | 79.3 ± 8.3 | [78.3; 80.3] | 104 (38.0) | [32.2; 44.0] | 170 (62.0) | [56.0; 67.8] | n. a. | n. a. | 53.5 ± 13.1 | [51.9; 55.1] |
|  | Ranibizumab 2.0 mg PRN | 273 | 78.3 ± 8.3 | [77.3; 79.3] | 117 (42.9) | [36.9; 49.0] | 156 (57.1) | [51.0; 63.1] | n. a. | n. a. | 53.5 ± 13.2 | [51.9; 55.1] |
| ^a^ The exact method of measuring baseline VA was not always explained in the sources and may vary. Therefore, direct comparisons of the VA results may not be reliable. ^b^ Missing values in OCEAN study: age: 14 patients; gender: 11; time since diagnosis: 199; baseline VA: 29. ^c^ Time since diagnosis of nAMD until first injection in OCEAN study. ^d^ Results converted to years, original data provided in days.  ^e^ Calculation of CIs based on an approximation assuming normal distribution; limited reliability of results due to high SD compared to mean.  ^f^ Missing values: Baseline VA: 0.5 mg ranibizumab group: 1 patient  ^g^ Missing values: Time since diagnosis: sham: 1 patient, 0.3 mg ranibizumab: 1 patient. ^h^ Only cohort 1 of the SAILOR trial included here, as this was blinded. Cohort 2 received open-label treatment. ^i^ Number (%) of males derived from females, 0 missings assumed.  ^k^ Number of females derived from percentage. ^m^ Data for the treatment-naïve patients in this treatment arm. ^n^ Data for the previously treated patients in this treatment arm. ^o^ Standard therapy: pegaptanib: 38 patients, verteporfin: 16, sham: 12. ^p^ Number (%) of females derived from males, 0 missings assumed.  ^q^ Percentage derived from number of patients. Abbreviations: CI: confidence interval; ETDRS: Early Treatment Diabetic Retinopathy Study; N: total number of patients; n: number of patients; n. a.: data not available; nAMD: neovascular age-related macular degeneration; PRN: pro re nata (as needed); q4: every 4 weeks; q6: every 6 weeks; q8: every 4 weeks for 3 months (3 doses) followed by dosing every 8 weeks; q16: every 4 months (quarterly); SD: standard deviation; VA: visual acuity. | | | | | | | | | | | | |
