# Supplementary material for: Demographics of patients receiving Intravitreal anti-VEGF treatment in real-world practice: healthcare research data versus randomized controlled trials
Source: BMC Ophthalmol. 2017 Jan 19;17:7. doi: 10.1186/s12886-017-0401-y (PMC5244516; doi:10.1186/s12886-017-0401-y)
Supplement: Additional file 4: Table S4. — Table of confidence intervals for medical history parameters in the indication neovascular age-related macular degeneration: results for the OCEAN study and for those selected randomized controlled trials for which comparable data were available. (DOCX 15 kb) [file 12886_2017_401_MOESM4_ESM.docx]

### **Additional File 4**

### **Table S4** Table of confidence intervals for medical history parameters in the indication neovascular age-related macular degeneration: results for the OCEAN study and for those selected randomized controlled trials for which comparable data were available.

| **Study** | **Treatment group** | **N** | **Patients with a medical history of** | | | | | | | |
| --- | --- | --- | --- | --- | --- | --- | --- | --- | --- | --- |
|  |  |  | **hypertension** | | **myocardial infarction** | | **stroke or apoplexy** | | **transient ischemic attack** | |
|  |  |  | n (%) | 95% CI (%) | n (%) | 95% CI (%) | n (%) | 95% CI (%) | n (%) | 95% CI (%) |
| **OCEAN** ^a^ | Ranibizumab 0.5 mg | 3614 | 804 (22.3) | [20.9; 23.6] | 198 (5.5) | [4.8; 6.3] | 146 (4.0) | [3.4; 4.7] | 2 (0.06) | [0.0; 0.2] |
| IVAN [36] ^b^ | Ranibizumab 0.5 mg | 314 | n. a. | n. a. | 24 (8) | [5.0; 11.2] | 7 (2) | [0.9; 4.5] | 20 (6) | [3.9; 9.7] |
|  | Bevacizumab 1.25 mg | 296 | n. a. | n. a. | 22 (7) | [4.7; 11.0] | 7 (2) | [1.0; 4.8] | 9 (3) | [1.4; 5.7] |
| CATT [38] | Ranibizumab 0.5 mg q4 | 146 | 102 (69.9) | [61.7; 77.2] | 15 (10.3) | [5.9; 16.4] | 6 (4.1) | [1.5; 8.7] | 8 (5.5) | [2.4; 10.5] |
|  | Bevacizumab 1.25 mg q4 | 135 | 93 (68.9) | [60.4; 73.6] | 16 (11.9) | [6.9; 18.5] | 7 (5.2) | [2.1; 10.4] | 12 (8.9) | [4.7; 15.0] |
|  | Ranibizumab 0.5 mg PRN | 287 | 195 (67.9) | [62.2; 73.3] | 28 (9.8) | [6.6; 13.8] | 22 (7.7) | [4.9; 11.4] | 11 (3.8) | [1.9; 6.8] |
|  | Bevacizumab 1.25 mg PRN | 270 | 196 (72.6) | [66.9; 77.8] | 33 (12.2) | [8.6; 16.7] | 16 (5.9) | [3.4; 9.5] | 17 (6.3) | [3.7; 9.9] |
|  | Ranibizumab 0.5 mg year 1 q4, year 2 PRN | 138 | 97 (70.3) | [61.9; 77.8] | 17 (12.3) | [7.3; 19.0] | 7 (5.1) | [2.1; 10.2] | 4 (2.9) | [0.8; 7.3] |
|  | Bevacizumab 1.25 mg year 1 q4, year 2 PRN | 131 | 84 (64.1) | [55.3; 72.3] | 19 (14.5) | [9.0; 21.7] | 9 (6.9) | [3.2; 12.6] | 11 (8.4) | [4.3; 14.5] |
| LUCAS [40] | Bevacizumab 1.25 mg | 213 | n. a. | n. a. | 12 (5.6) | [2.9; 9.6] | 11 (5.2) ^c^ | [2.6; 9.1] | 11 (5.2) | [2.6; 9.1] |
|  | Ranibizumab 0.5 mg | 218 | n. a. | n. a. | 26 (11.9) | [7.9; 17.0] | 11 (5.0) ^c^ | [2.6; 8.9] | 12 (5.5) | [2.9; 9.4] |
| GEFAL [41] | Bevacizumab 1.25 mg | 191 | 119 (62.3) | [55.0; 69.2] | 10 (5.2) | [2.5; 9.4] | 7 (3.7) | [1.5; 7.4] | 1 (0.5) | [0.0; 2.9] |
|  | Ranibizumab 0.5 mg | 183 | 94 (51.4) | [43.9; 58.8] | 3 (1.6) | [0.3; 4.7] | 3 (1.6) | [0.3; 4.7] | 0 (0.0) | [0.0; 2.0] |
| ^a^ Missing values in OCEAN study: medical history: 27 patients. ^b^ Missing values: Transient ischemic attack: ranibizumab: 9 patients, bevacizumab: 11; stroke: bevacizumab: 1 patient. ^c^ Stroke defined as “cerebrovascular infarction” in this study. Abbreviations: CI: confidence interval; N: total number of patients; n: number of patients; n. a.: data not available; nAMD: neovascular age-related macular degeneration; PRN: pro re nata (as needed); q4: every 4 weeks. | | | | | | | | | | |
