# Supplementary material for: Demographics of patients receiving Intravitreal anti-VEGF treatment in real-world practice: healthcare research data versus randomized controlled trials
Source: BMC Ophthalmol. 2017 Jan 19;17:7. doi: 10.1186/s12886-017-0401-y (PMC5244516; doi:10.1186/s12886-017-0401-y)
Supplement: Additional file 5: Table S5. — Table of confidence intervals for baseline demographic characteristics (age, gender, time since diagnosis, baseline visual acuity) in the indication diabetic macular oedema: results for the OCEAN study and for selected randomized controlled trials. (DOCX 20 kb) [file 12886_2017_401_MOESM5_ESM.docx]

### **Additional File 5**

### **Table S5** Table of confidence intervals for baseline demographic characteristics (age, gender, time since diagnosis, baseline visual acuity) in the indication diabetic macular oedema: results for the OCEAN study and for selected randomized controlled trials.

| **Study** | **Treatment group** | **N** | **Age** | | **Gender** | | | | **Time since diagnosis of DME** | | **Baseline VA** | |
| --- | --- | --- | --- | --- | --- | --- | --- | --- | --- | --- | --- | --- |
|  |  |  | Mean ± SD (years) | 95% CI (years) | Males,  n (%) | Males,  95% CI (%) | Females  n (%) | Females, 95% CI (%) | Mean ±SD (years) | 95% CI (years) | ETDRS letters analogue (mean ± SD) ^a^ | 95% CI (letters) |
| **OCEAN** ^b^ | Ranibizumab 0.5 mg | 1211 | 67.6 ± 10.9 | [67.0; 68.2] | 698 (57.6) | [54.8; 60.4] | 507 (41.9) | [39.1; 44.7] | 0.68 ± 1.63 ^c, d^ | [0.59; 0.77] ^e^ | 60.6 ± 15.5 | [59.7; 61.5] |
| DRCR.net Protocol J [43] | Sham injection + laser | 123 | n. a. | n. a. | 79 (64.2) ^f^ | [55.1; 72.7] | 44 (36) | [27.3; 44.9] | n. a. | n. a. | n. a. | n. a. |
|  | Ranibizumab + laser | 113 | n. a. | n. a. | 65 (57.5) ^f^ | [47.9; 66.8] | 48 (42) | [33.2; 52.1] | n. a. | n. a. | n. a. | n. a. |
|  | Triamcinolone + laser | 109 | n. a. | n. a. | 65 (59.6) ^f^ | [49.8; 68.9] | 44 (40) | [31.1; 50.2] | n. a. | n. a. | n. a. | n. a. |
| DRCR.net Protocol I [44] | Sham injection + prompt laser | 293 | n. a. | n. a. | 170 (58.0) ^f^ | [52.1; 63.7] | 123 (42) | [36.3; 47.9] | n. a. | n. a. | n. a. | n. a. |
|  | Ranibizumab + prompt laser | 187 | n. a. | n. a. | 102 (54.5) ^f^ | [47.1; 61.8] | 85 (45) | [38.2; 52.9] | n. a. | n. a. | n. a. | n. a. |
|  | Ranibizumab + deferred laser | 188 | n. a. | n. a. | 110 (58.5) ^f^ | [51.1; 65.6] | 78 (41) | [34.4; 48.9] | n. a. | n. a. | n. a. | n. a. |
|  | Triamcinolone + prompt laser | 186 | n. a. | n. a. | 100 (53.8) ^f^ | [46.3; 61.1] | 86 (46) | [38.9; 53.7] | n. a. | n. a. | n. a. | n. a. |
| BOLT [45] | Bevacizumab | 42 | 64.9 ± 9.4 | [62.1; 67.7] | 30 (71.4) ^g^ | [55.4; 84.3] | 12 (28.6) ^g^ | [15.7; 44.6] | n. a. | n. a. | 55.7 ± 9.7 | [52.8; 58.6] |
|  | Laser | 38 | 63.5 ± 8.1 | [60.9; 66.1] | 25 (65.8) ^g^ | [48.7; 80.4] | 13 (34.2) ^g^ | [19.6; 51.4] | n. a. | n. a. | 54.6 ± 8.6 | [51.9; 57.3] |
| RISE [46] ^h^ | Sham injections | 127 | 61.8 ± 9.8 | [60.1; 63.5] | 74 (58.3) | [49.2; 67.0] | 53 (41.7) ^i^ | [33.1; 50.8] | 2.3 ± 3.0 ^k^ | [1.77; 2.83] ^e^ | 57.2 ± 11.11 | [55.3; 59.1] |
|  | Ranibizumab 0.3 mg | 125 | 61.7 ± 8.9 | [60.1; 63.3] | 73 (58.4) | [49.3; 67.2] | 52 (41.6) ^i^ | [32.9; 50.8] | 2.1 ± 2.2 ^k^ | [1.71; 2.49] | 54.7 ± 12.6 | [52.5; 56.9] |
|  | Ranibizumab 0.5 mg | 125 | 62.8 ± 10.0 | [61.0; 64.6] | 65 (52.0) | [42.9; 61.0] | 60 (48.0) ^i^ | [39.0; 57.1] | 2.1 ± 2.1 ^k^ | [1.74; 2.46] | 56.9 ± 11.6 | [54.9; 58.9] |
| RIDE [46] ^m^ | Sham injections | 130 | 63.5 ± 10.8 | [61.6; 65.4] | 66 (50.8) | [41.9; 59.6] | 64 (49.2) ^i^ | [40.4; 58.1] | 2.4 ± 3.2 ^k^ | [1.84; 2.96] ^e^ | 57.3 ± 11.2 | [55.4; 59.2] |
|  | Ranibizumab 0.3 mg | 125 | 62.7 ± 11.1 | [60.8; 64.6] | 73 (58.4) | [49.3; 67.2] | 52 (41.6) ^i^ | [32.9; 50.8] | 1.6 ± 2.0 ^k^ | [1.25; 1.95] ^e^ | 57.5 ± 11.6 | [55.5; 59.5] |
|  | Ranibizumab 0.5 mg | 127 | 61.8 ± 10.1 | [60.0; 63.6] | 80 (63.0) | [54.0; 71.4] | 47 (37.0) ^i^ | [28.6; 46.0] | 1.9 ± 2.4 ^k^ | [1.46; 2.34] ^e^ | 56.9 ± 11.8 | [54.8; 59.0] |
| RESTORE [47] | Ranibizumab 0.5 mg + sham laser | 116 | 62.9 ± 9.29 | [61.2; 64.6] | 73 (62.9) | [53.5; 71.7] | 43 (37.1) | [28.3; 46.5] | 1.80 ± 1.98 | [1.44; 2.16] | 64.8 ± 10.11 | [63.0; 66.6] |
|  | Ranibizumab 0.5 mg + laser | 118 | 64.0 ± 8.15 | [62.5; 65.5] | 70 (59.3) | [49.9; 68.3] | 48 (40.7) | [31.7; 50.1] | 1.99 ± 3.14 | [1.41; 2.57] ^e^ | 63.4 ± 9.99 | [61.6; 65.2] |
|  | Laser + sham injection | 111 | 63.5 ± 8.81 | [61.9; 65.1] | 58 (52.3) | [42.6; 61.8] | 53 (47.7) | [38.2; 57.4] | 1.58 ± 1.96 | [1.27; 1.89] ^e^ | 62.4 ± 11.11 | [60.3; 64.5] |
| VISTA [48] | Laser photo-coagulation + sham injection | 154 | 61.7 ± 8.7 | [60.3; 63.1] | 85 (55.2) ^f^ | [47.0; 63.2] | 69 (44.8) | [36.8; 53.2] | n. a. | n. a. | 59.7 ± 10.9 | [58.0; 61.4] |
|  | Aflibercept 2 mg 2q4 | 154 | 62.0 ± 11.2 | [60.2; 63.8] | 87 (56.5) ^f^ | [48.3; 64.5] | 67 (43.5) | [35.6; 51.7] | n. a. | n. a. | 58.9 ± 10.8 | [57.2; 60.6] |
|  | Aflibercept 2 mg 2q8 | 151 | 63.1 ± 9.4 | [61.6; 64.6] | 78 (51.7) ^f^ | [43.4; 59.9] | 73 (48.3) | [40.2; 56.6] | n. a. | n. a. | 59.4 ± 10.9 | [57.7; 61.1] |
| VIVID [48] | Laser photo-coagulation + sham injection | 132 | 63.9 ± 8.6 | [62.4; 65.4] | 78 (59.1) ^f^ | [50.2; 67.6] | 54 (40.9) | [32.4; 49.8] | n. a. | n. a. | 60.8 ± 10.6 | [59.0; 62.6] |
|  | Aflibercept 2 mg 2q4 | 136 | 62.6 ± 8.6 | [61.2; 64.0] | 83 (61.0) ^f^ | [52.3; 69.3] | 53 (39.0) | [30.7; 47.7] | n. a. | n. a. | 60.8 ± 10.7 | [59.0; 62.6] |
|  | Aflibercept 2 mg 2q8 | 135 | 64.2 ± 7.8 | [62.9; 65.5] | 88 (65.2) ^f^ | [56.5; 73.2] | 47 (34.8) | [26.8; 43.5] | n. a. | n. a. | 58.8 ± 11.2 | [56.9; 60.7] |
| DRCR.net Protocol T [49] | Aflibercept | 224 | 60 ± 10 | [58.7; 61.3] | 114 (50.9) ^f^ | [44.2; 57.6] | 110 (49) | [42.4; 55.9] | n. a. | n. a. | n. a. | n. a. |
|  | Bevacizumab | 218 | 62 ± 10 | [60.7; 63.3] | 115 (52.8) ^f^ | [45.9; 59.5] | 103 (47) | [40.5; 54.1] | n. a. | n. a. | n. a. | n. a. |
|  | Ranibizumab | 218 | 60 ± 11 | [58.5; 61.5] | 124 (56.9) ^f^ | [50.0; 63.6] | 94 (43) | [36.5; 50.0] | n. a. | n. a. | n. a. | n. a. |
| ^a^ The exact method of measuring baseline VA was not always explained in the sources and may vary. Therefore, direct comparisons of the VA results may not be reliable. ^b^ Missing values in OCEAN study: age: 9 patients; gender: 6; BMI: 87; time since diagnosis of DME: 65; baseline VA: 8. ^c^ Time since diagnosis of DME until first injection in OCEAN study. ^d^ Results converted to years, original data provided in days. ^e^ Calculation of CIs based on an approximation assuming normal distribution; limited reliability of results due to high SD compared to mean. ^f^ Number (%) of males derived from females, 0 missings assumed. ^g^ Percentage derived from number of patients.  ^h^ Missing values in RISE study (in sham, 0.3 mg, 0.5 mg group, respectively): time since diagnosis of DME: 0, 1, 2.  ^i^ Number (%) of females derived from males, 0 missings assumed. ^k^ Defined as mean time from first known clinically significant macular oedema to randomization. ^m^ Missing values in RIDE study (in sham, 0.3 mg, 0.5 mg group, respectively): time since diagnosis of DME: 0, 0, 1. Abbreviations: CI: confidence interval; 2q4: every 4 weeks from baseline to week 48; 2q8: every 4 weeks from baseline to week 16 (5 doses) followed by dosing every 8 weeks through week 48; DME: diabetic macular oedema; DRCR.net: Diabetic Retinopathy Clinical Research Network; ETDRS: Early Treatment Diabetic Retinopathy Study; N: total number of patients; n: number of patients; n. a.: data not available; SD: standard deviation; VA: visual acuity. | | | | | | | | | | | | |
