# Supplementary material for: Demographics of patients receiving Intravitreal anti-VEGF treatment in real-world practice: healthcare research data versus randomized controlled trials
Source: BMC Ophthalmol. 2017 Jan 19;17:7. doi: 10.1186/s12886-017-0401-y (PMC5244516; doi:10.1186/s12886-017-0401-y)
Supplement: Additional file 6: Table S6. — Table of confidence intervals for additional baseline demographic parameters (BMI, diabetes type, HbA1c) in the indication diabetic macular oedema: results for the OCEAN study and for selected randomized controlled trials. (DOCX 18 kb) [file 12886_2017_401_MOESM6_ESM.docx]

### **Additional File 6**

### **Table S6** Table of confidence intervals for additional baseline demographic parameters (BMI, diabetes type, HbA1c) in the indication diabetic macular oedema: results for the OCEAN study and for selected randomized controlled trials.

| **Study** | **Treatment group** | **N** | **BMI** | | **Diabetes type** | | | | **HbA1c** | |
| --- | --- | --- | --- | --- | --- | --- | --- | --- | --- | --- |
|  |  |  | Mean ± SD (kg/m^2^) | 95% CI (kg/m^2^) | Type I,  n (%) | Type I,  95% CI (%) | Type II,  n (%) | Type II,  95% CI (%) | Mean ± SD (%) | 95% CI (%) |
| **OCEAN** ^a^ | Ranibizumab 0.5 mg | 1211 | 29.3 ± 5.2 | [29.0; 29.6] | 116 (9.6) | [8.0; 11.4] | 936 (77.3) | [74.8; 79.6] | 7.5 ± 1.3 | [7.41; 7.59] |
| DRCR.net Protocol J [43] ^b^ | Sham injection + laser | 123 | n. a. | n. a. | 20 (16) | [10.2; 24.0] | 101 (82) | [74.2; 88.4] | n. a. | n. a. |
|  | Ranibizumab + laser | 113 | n. a. | n. a. | 13 (12) | [6.3; 18.9] | 93 (82) | [74.0; 88.8] | n. a. | n. a. |
|  | Triamcinolone + laser | 109 | n. a. | n. a. | 12 (11) | [5.8; 18.4] | 95 (87) | [79.4; 92.8] | n. a. | n. a. |
| DRCR.net Protocol I [44] ^c^ | Sham injection + prompt laser | 293 | n. a. | n. a. | 25 (9) | [5.6; 12.3] | 260 (89) | [84.6; 92.1] | n. a. | n. a. |
|  | Ranibizumab + prompt laser | 187 | n. a. | n. a. | 11 (6) | [3.0; 10.3] | 172 (92) | [87.1; 95.4] | n. a. | n. a. |
|  | Ranibizumab + deferred laser | 188 | n. a. | n. a. | 15 (8) | [4.5; 12.8] | 170 (90) | [85.3; 94.2] | n. a. | n. a. |
|  | Triamcinolone + prompt laser | 186 | n. a. | n. a. | 14 (8) | [4.2; 12.3] | 166 (89) | [83.9; 93.3] | n. a. | n. a. |
| BOLT [45] | Bevacizumab | 42 | n. a. | n. a. | 4 (9.5) ^d^ | [2.7; 22.6] | 38 (90.5) ^d^ | [77.4; 97.3] | 7.6 ± 1.4 | [7.18; 8.02] |
|  | Laser | 38 | n. a. | n. a. | 4 (10.5) ^d^ | [2.9; 24.8] | 34 (89.5) ^d^ | [75.2; 97.1] | 7.5 ± 1.2 | [7.12; 7.88] |
| RISE [46] ^e^ | Sham injections | 127 | 31.4 ± 7.1 | [30.2; 32.6] | n. a. | n. a. | n. a. | n. a. | 7.7 ± 1.5 | [7.43; 7.97] |
|  | Ranibizumab 0.3 mg | 125 | 32.3 ± 6.8 | [31.1; 33.5] | n. a. | n. a. | n. a. | n. a. | 7.7 ± 1.5 | [7.43; 7.97] |
|  | Ranibizumab 0.5 mg | 125 | 32.9 ± 8.5 | [31.4; 34.4] | n. a. | n. a. | n. a. | n. a. | 7.7 ± 1.4 | [7.45; 7.95] |
| RIDE [46] ^f^ | Sham injections | 130 | 32.3 ± 8.9 | [30.8; 33.8] | n. a. | n. a. | n. a. | n. a. | 7.6 ± 1.4 | [7.35; 7.85] |
|  | Ranibizumab 0.3 mg | 125 | 32.3 ± 8.6 | [30.8; 33.8] | n. a. | n. a. | n. a. | n. a. | 7.6 ± 1.3 | [7.37; 7.83] |
|  | Ranibizumab 0.5 mg | 127 | 31.3 ± 7.2 | [30.0; 32.6] | n. a. | n. a. | n. a. | n. a. | 7.6 ± 1.5 | [7.33; 7.87] |
| RESTORE [47] | Ranibizumab 0.5 mg + sham laser | 116 | n. a. | n. a. | 13 (11.2) | [6.1; 18.4] | 103 (88.8) | [81.6; 93.9] | n. a. | n. a. |
|  | Ranibizumab 0.5 mg + laser | 118 | n. a. | n. a. | 15 (12.7) | [7.3; 20.1] | 102 (86.4) | [78.9; 92.1] | n. a. | n. a. |
|  | Laser + sham injection | 111 | n. a. | n. a. | 13 (11.7) | [6.4; 19.2] | 97 (87.4) | [79.7; 92.9] | n. a. | n. a. |
| VISTA [48] | Laser photo-coagulation + sham injection | 154 | n. a. | n. a. | n. a. | n. a. | n. a. | n. a. | 7.6 ± 1.7 | [7.33; 7.87] |
|  | Aflibercept 2 mg 2q4 | 154 | n. a. | n. a. | n. a. | n. a. | n. a. | n. a. | 7.9 ± 1.6 | [7.65; 8.15] |
|  | Aflibercept 2 mg 2q8 | 151 | n. a. | n. a. | n. a. | n. a. | n. a. | n. a. | 7.9 ± 1.6 | [7.64; 8.16] |
| VIVID [48] | Laser photo-coagulation + sham injection | 132 | n. a. | n. a. | n. a. | n. a. | n. a. | n. a. | 7.7 ± 1.3 | [7.48; 7.92] |
|  | Aflibercept 2 mg 2q4 | 136 | n. a. | n. a. | n. a. | n. a. | n. a. | n. a. | 7.8 ± 1.5 | [7.55; 8.05] |
|  | Aflibercept 2 mg 2q8 | 135 | n. a. | n. a. | n. a. | n. a. | n. a. | n. a. | 7.7 ± 1.4 | [7.46; 7.94] |
| DRCR.net Protocol T [49] ^g^ | Aflibercept | 224 | n. a. | n. a. | 22 (10) | [6.3; 14.5] | 196 (88) | [82.4; 91.5] | n. a. | n. a. |
|  | Bevacizumab | 218 | n. a. | n. a. | 12 (6) | [2.9; 9.4] | 205 (94) | [90.0; 96.8] | n. a. | n. a. |
|  | Ranibizumab | 218 | n. a. | n. a. | 16 (7) | [4.3; 11.7] | 196 (90) | [85.1; 93.6] | n. a. | n. a. |
| ^a^ Missing values in OCEAN study: BMI: 87 patients; diabetes type: 69; HbA1c: 491. ^b^ Missing values in DCRC.net Protocol J (sham, ranibizumab, triamcinolone group, respectively): HbA1c: 3, 10, 6 patient(s). ^c^ Missing values in DCRC.net Protocol I (sham, ranibizumab+prompt laser, ranibizumab+deferred laser, triamcinolone group, respectively): HbA1c: 17, 3, 7, 8 patient(s). ^d^ Percentage derived from number of patients. ^e^ Missing values in RISE study (in sham, 0.3 mg, 0.5 mg group, respectively): BMI: 3, 3, 1 patient(s); HbA1c: 4, 5, 5 patients. ^f^ Missing values in RIDE study (in sham, 0.3 mg, 0.5 mg group, respectively): BMI: 2, 0, 1 patient(s); HbA1c: 5, 5, 4 patients. ^g^ Missing values in DCRC.net Protocol T (aflibercept, bevacizumab, ranibizumab group, respectively): diabetes type: 6, 1, 6 patients; HbA1c: 5, 0, 1 patient(s); BMI: 17, 22, 23 patients. Abbreviations: CI: confidence interval; 2q4: every 4 weeks from baseline to week 48; 2q8: every 4 weeks from baseline to week 16 (5 doses) followed by dosing every 8 weeks through week 48; BMI: body mass index; DME: diabetic macular oedema; DRCR.net: Diabetic Retinopathy Clinical Research Network; HbA1c: glycated haemoglobin (type A1c); N: total number of patients; n: number of patients; n. a.: data not available; SD: standard deviation. | | | | | | | | | | |
