# Supplementary material for: Demographics of patients receiving Intravitreal anti-VEGF treatment in real-world practice: healthcare research data versus randomized controlled trials
Source: BMC Ophthalmol. 2017 Jan 19;17:7. doi: 10.1186/s12886-017-0401-y (PMC5244516; doi:10.1186/s12886-017-0401-y)
Supplement: Additional file 7: Table S7. — Table of confidence intervals for baseline demographic characteristics (age, gender, time since diagnosis, baseline visual acuity) in the indications branch and central retinal vein occlusion: results for the OCEAN study and for selected randomized controlled trials. (DOCX 20 kb) [file 12886_2017_401_MOESM7_ESM.docx]

### **Additional File 7**

### **Table S7** Table of confidence intervals for baseline demographic characteristics (age, gender, time since diagnosis, baseline visual acuity) in the indications branch and central retinal vein occlusion: results for the OCEAN study and for selected randomized controlled trials.

| **Study** | **Treatment group** | **N** | **Age** | | **Gender** | | | | **Time since diagnosis of RVO** | | **Baseline VA** | | |
| --- | --- | --- | --- | --- | --- | --- | --- | --- | --- | --- | --- | --- | --- |
|  |  |  | Mean ± SD (years) | 95% CI (years) | Males,  n (%) | Males,  95% CI (%) | Females  n (%) | Females, 95% CI (%) | Mean ±SD (months) | 95% CI (months) | ETDRS letters analogue (mean ± SD) ^a^ | 95% CI (letters) | |
| **Indication BRVO** | |  |  |  |  |  |  |  |  |  |  |  | |
| **OCEAN** ^b^ | Ranibizumab 0.5 mg | 204 | 71.2 ± 10.0 | [69.8; 72.6] | 85 (41.7) | [34.8; 48.8] | 119 (58.3) | [51.2; 65.2] | 6.30 ± 16.35 ^c,d^ | [4.04; 8.56] ^e^ | 55.9 ± 20.9 | [53.0; 58.8] | |
| BRAVO [50] | Sham injections  (6 months) | 132 | 65.2 ± 12.7 | [63.0; 67.4] | 74 (56.1) | [47.2; 64.7] | 58 (43.9) | [35.3; 52.8] | 3.7 ± 3.7 | [3.07; 4.33] ^e^ | 54.7 ± 12.2 | [52.6; 56.8] | |
|  | Ranibizumab 0.3 mg | 134 | 66.6 ± 11.2 | [64.7; 68.5] | 67 (50.0) | [41.3; 58.8] | 67 (50.0) | [41.3; 58.8] | 3.6 ± 4.1 | [2.91; 4.29] ^e^ | 56.0 ± 12.1 | [54.0; 58.0] | |
|  | Ranibizumab 0.5 mg | 131 | 67.5 ± 11.8 | [65.5; 69.5] | 71 (54.2) | [45.3; 62.9] | 60 (45.8) | [37.1; 54.7] | 3.3 ± 3.1 | [2.77; 3.83] ^e^ | 53.0 ± 12.5 | [50.9; 55.1] | |
| VIBRANT [51] | Laser | 90 | 63.9 ± 11.4 | [61.5; 66.3] | 54 (60.0) ^f^ | [49.1; 70.2] | 36 (40.0) | [29.8; 50.9] | n. a. | n. a. | 57.7 ± 11.3 | [55.4; 60.0] | |
|  | Aflibercept 2 mg | 91 | 67.0 ± 10.4 | [64.9; 69.1] | 44 (48.4) ^f^ | [37.7; 59.1] | 47 (51.6) | [40.9; 62.3] | n. a. | n. a. | 58.6 ± 11.4 | [56.3; 60.9] | |
| **Indication CRVO** | |  |  |  |  |  |  |  |  |  |  |  | |
| **OCEAN** ^b^ | Ranibizumab  0.5 mg | 121 | 70.3 ± 11.5 | [68.3; 72.3] | 57 (47.1) | [38.0; 56.4] | 64 (52.9) | [43.6; 62.0] | 3.78 ± 6.49 ^c,d^ | [2.61; 4.95] ^e^ | 43.7 ± 25.0 | [39.2; 48.2] | |
| CRUISE [52] | Sham injections | 130 | 65.4 ± 13.1 | [63.1; 67.7] | 72 (55.4) | [46.4; 64.1] | 58 (44.6) | [35.9; 53.6] | 2.9 ± 2.9 | [2.40; 3.40] ^e^ | 49.2 ± 14.7 | [46.7; 51.7] | |
|  | Ranibizumab 0.3 mg | 132 | 69.7 ± 11.6 | [67.7; 71.7] | 71 (53.8) | [44.9; 62.5] | 61 (46.2) | [37.5; 55.1] | 3.6 ± 3.2 | [3.05; 4.15] ^e^ | 47.4 ± 14.8 | [44.9; 49.9] | |
|  | Ranibizumab 0.5 mg | 130 | 67.6 ± 12.4 | [65.5; 69.7] | 80 (61.5) | [52.6; 69.9] | 50 (38.5) | [30.1; 47.4] | 3.3 ± 3.7 | [2.66; 3.94] ^e^ | 48.1 ± 14.6 | [45.6; 50.6] | |
| COPERNICUS [53] ^g^ | Aflibercept 2 mg 2q4, then PRN | 114 | 65.5 ± 13.57 | [63.0; 68.0] | 69 (61) | [50.9; 69.6] | 45 (39) | [30.5; 49.1] | 2.73 ± 3.09 | [2.16; 3.30] ^e^ | 50.7 ± 13.9 | [48.1; 53.3] | |
|  | Sham injections 2q4, then Aflibercept 2 mg PRN | 73 | 67.5 ± 14.29 | [64.2; 70.8] | 38 (52) | [40.0; 63.9] | 35 (48) | [36.1; 60.0] | 1.88 ± 2.19 | [1.38; 2.38] ^e^ | 48.9 ± 14.4 | [45.6; 52.2] | |
| GALILEO [54] | VEGF Trap-Eye (Aflibercept) 2q4 | 103 | 59.9 ± 12.4 | [57.5; 62.3] | 58 (56.3) | [46.2; 66.1] | 45 (43.7) | [33.9; 53.8] | 2.6 ± 2.9 ^d^ | [2.04; 3.16] ^e^ | 53.6 ± 15.8 | [50.5; 56.7] | |
|  | Sham injections | 68 | 63.8 ± 13.3 | [60.6; 67.0] | 37 (54.4) | [41.9; 66.6] | 31 (45.6) | [33.5; 58.1] | 2.9 ± 2.6 ^d^ | [2.28; 3.52] ^e^ | 50.9 ± 15.4 | | [47.2; 54.6] |
| ^a^ The exact method of measuring baseline VA was not always explained in the sources and may vary. Therefore, direct comparisons of the VA results may not be reliable. ^b^ Missing values in OCEAN study: time since first diagnosis of RVO: 3 in BRVO, 3 in CRVO; baseline VA: 1 in BRVO, 3 in CRVO. ^c^ Time since diagnosis of RVO until first injection in OCEAN study. ^d^ Results converted to months, original data provided in days.  ^e^ Calculation of CIs based on an approximation assuming normal distribution; limited reliability of results due to high SD compared to mean. ^f^ Number (%) of males derived from females, 0 missings assumed. ^g^ Missing values in COPERNICUS study: Time since first diagnosis of RVO: 1 patient (in treatment group “Aflibercept 2 mg 2q4, then PRN”) Abbreviations: CI: confidence interval; 2q4: every 4 weeks for 24 weeks; BRVO: branch retinal vein occlusion; CRVO: central retinal vein occlusion; ETDRS: Early Treatment Diabetic Retinopathy Study; N: total number of patients; n: number of patients; n. a.: data not available; PRN: pro re nata (as needed); RVO: retinal vein occlusion; SD: standard deviation; VA: visual acuity. | | | | | | | | | | | | | |
